# Candidate SNP Markers of Familial and Sporadic Alzheimer's Diseases Are Predicted by a Significant Change in the Affinity of TATA-Binding Protein for Human Gene Promoters

**DOI:** 10.3389/fnagi.2017.00231

**Published:** 2017-07-20

**Authors:** Petr Ponomarenko, Irina Chadaeva, Dmitry A. Rasskazov, Ekaterina Sharypova, Elena V. Kashina, Irina Drachkova, Dmitry Zhechev, Mikhail P. Ponomarenko, Ludmila K. Savinkova, Nikolay Kolchanov

**Affiliations:** ^1^Children's Hospital Los Angeles, University of Southern California Los Angeles, CA, United States; ^2^Division for System Biology, Institute of Cytology and Genetics of Siberian Branch of Russian Academy of Sciences Novosibirsk, Russia; ^3^Faculty of Natural Sciences, Novosibirsk State University Novosibirsk, Russia

**Keywords:** gene, promoter, TATA-binding protein, TBP-binding site, single nucleotide polymorphism, expression change, Alzheimer's disease, SNP marker

## Abstract

While year after year, conditions, quality, and duration of human lives have been improving due to the progress in science, technology, education, and medicine, only eight diseases have been increasing in prevalence and shortening human lives because of premature deaths according to the retrospective official review on the state of US health, 1990-2010. These diseases are kidney cancer, chronic kidney diseases, liver cancer, diabetes, drug addiction, poisoning cases, consequences of falls, and Alzheimer's disease (AD) as one of the leading pathologies. There are familial AD of hereditary nature (~4% of cases) and sporadic AD of unclear etiology (remaining ~96% of cases; i.e., non-familial AD). Therefore, sporadic AD is no longer a purely medical problem, but rather a social challenge when someone asks oneself: “What can I do in my own adulthood to reduce the risk of sporadic AD at my old age to save the years of my lifespan from the destruction caused by it?” Here, we combine two computational approaches for regulatory SNPs: Web service SNP_TATA_Comparator for sequence analysis and a PubMed-based keyword search for articles on the biochemical markers of diseases. Our purpose was to try to find answers to the question: “What can be done in adulthood to reduce the risk of sporadic AD in old age to prevent the lifespan reduction caused by it?” As a result, we found 89 candidate SNP markers of familial and sporadic AD (e.g., rs562962093 is associated with sporadic AD in the elderly as a complication of stroke in adulthood, where natural marine diets can reduce risks of both diseases in case of the minor allele of this SNP). In addition, rs768454929, and rs761695685 correlate with sporadic AD as a comorbidity of short stature, where maximizing stature in childhood and adolescence as an integral indicator of health can minimize (or even eliminate) the risk of sporadic AD in the elderly. After validation by clinical protocols, these candidate SNP markers may become interesting to the general population [may help to choose a lifestyle (in childhood, adolescence, and adulthood) that can reduce the risks of sporadic AD, its comorbidities, and complications in the elderly].

## Introduction

While year after year, conditions, quality, and duration of human lives have been improving due to the progress in science, technology, education, and medicine, only eight diseases have been increasing in prevalence and shortening human lives because of premature deaths according to the retrospective official review on the state of US health, 1990-2010 (Murray et al., 2013). These diseases are kidney cancer, chronic kidney diseases, liver cancer, diabetes, drug addiction, poisoning cases, consequences of falls, and Alzheimer's disease (AD) as one of the leading pathologies, which is also first among dementias in terms of prevalence (Brookmeyer et al., 2007). According to Harvey et al. (2003), there are two forms of AD, namely: familial AD of hereditary nature (~4% of the total number of cases; probability varies from 40 to 70% from family to family) and sporadic AD of unclear etiology (remaining ~96% of cases; i.e., non-familial AD). Therefore, sporadic AD is no longer a purely medical problem and has already become a social challenge where many people ask themselves: “What can I do in my own adulthood to save the years of my lifespan from the destruction caused by sporadic AD at my old age?” This desire of the general population to actively maintain their own health is one of the main reasons behind postgenomic predictive preventive personalized medicine (Trovato, 2014). This new branch of medicine uses biomedical SNP markers, which enable discrimination of individual genomes of patients with a given pathology from the reference human genome as the norm.

Identification of SNPs on the whole-genome scale is one of the biggest modern scientific projects: “1,000 Genomes” (Colonna et al., 2014). The current results of this project can be found in the dbSNP database (Sherry et al., 2001). This database is an inherent part of the reference human genome and contains the ancestral alleles of all SNPs, whereas the human variome represents their minor alleles. These data are publicly available due to the Web service UCSC Genome Browser (Haeussler et al., 2015). Clinical comparison between a cohort of patients with a given disease and healthy volunteers (as a control) enables identification of SNPs whose alleles significantly discriminate them from one another and became SNP markers of this disease; this result, however, is very costly and labor-intensive (Yoo et al., 2015). Computer-based analysis of 8.58 billion possible human whole-genome SNPs, predictions, experimental data, and the related clinical observations that are accumulated in database dbWGFP (Wu et al., 2016) may accelerate and direct the clinical search for biomedical SNP markers (Ponomarenko et al., 2001). Many public Web services (for review, see Deplancke et al., 2016) facilitate this computer-based search for candidate SNP markers by a variety of methods and approaches, which yield better results for some types of SNP and diseases and worse results for others; thus, a combination of different services should improve the overall quality of results. Currently, SNPs located in protein-coding gene regions are studied most thoroughly because of the invariant types of disruption in both structure and function of the altered protein (Amberger et al., 2015), and these aberrations are often fatal and uncorrectable by medication. In contrast, the disruptions caused by another sort of SNPs, those located in regulatory gene regions, are correctable by medication because only amounts of the protein product of these genes vary, whereas both structure and function of these proteins remain normal. These regulatory SNPs are studied the least because their manifestations vary from cell to cell, from tissue to tissue, from individual to individual, from subpopulation to subpopulation (Amberger et al., 2015). The majority of the known regulatory SNP markers alter the binding sites for TATA-binding protein (TBP) because of their fixed locations within the narrow region [−70; −20] upstream of the transcription start site of a protein-coding transcript (Ponomarenko et al., 2013). This sort of the SNP markers has been easier to detect due to the positive correlation between the expression level of the human gene containing them and the affinity of TBP for the promoter of this gene (Mogno et al., 2010). This especially high importance of the TBP binding to the promoter can be attributed to the fact that this is the very first obligatory molecular event in the course of the transcription initiation in eukaryotes (Muller et al., 2001; Martianov et al., 2002; Ponomarenko et al., 2013).

In our previous works, we created the public Web service SNP_TATA_Comparator^1^ for estimation of statistical significance of the SNP-caused alterations of a given TBP-binding site in the binary terms of either under- or overexpression of the gene being studied (Ponomarenko et al., 2015). Next, we verified our predictions on this subject by our own experiments *in vitro* under either real-time (Arkova et al., 2014), equilibrium (Savinkova et al., 2013), and non-equilibrium (Drachkova et al., 2014) conditions as well as using independent data from over 60 experiments by others (for review, see Ponomarenko et al., 2010). Accordingly, we have already applied our Web service (Ponomarenko et al., 2015) to prediction of candidate SNP markers of the following medical conditions: complications of hereditary diseases in obesity (Arkova et al., 2015), autoimmune comorbidities of these hereditary diseases (Ponomarenko M. et al., 2016), circadian rhythm disorders (Ponomarenko V. et al., 2016), aggressiveness as a complication of human diseases (Chadaeva et al., 2016), and resistance to antitumor chemotherapy (Turnaev et al., 2016) (for review, see Ponomarenko et al., 2017).

In this work, we extended the use of our Web service to unannotated SNPs near known biomedical SNP markers in TBP-binding sites of promoters in human genes, associated with hereditary diseases, which were taken from our previous review (Ponomarenko et al., 2015). Our purpose was to try to find answers to the question: “What can be done in adulthood to reduce the risk of sporadic AD in old age to prevent the lifespan reduction caused by it?” within the framework of postgenomic predictive preventive personalized medicine (Trovato, 2014). We also analyzed unannotated SNPs located within the core promoters of a human gene associated with familial AD. Among the numerous candidates, we selected 89 candidate SNP markers of familial and sporadic AD. Validation of these candidate markers by clinical protocols can make these SNPs interesting to the general population [e.g., may help to choose a lifestyle (in childhood, adolescence, and adulthood) that can reduce the risks of sporadic AD, its comorbidities, and complications in the elderly] within the framework of postgenomic predictive preventive personalized medicine (Trovato, 2014).

## Materials and methods

### DNA sequences

**Figure 1:**
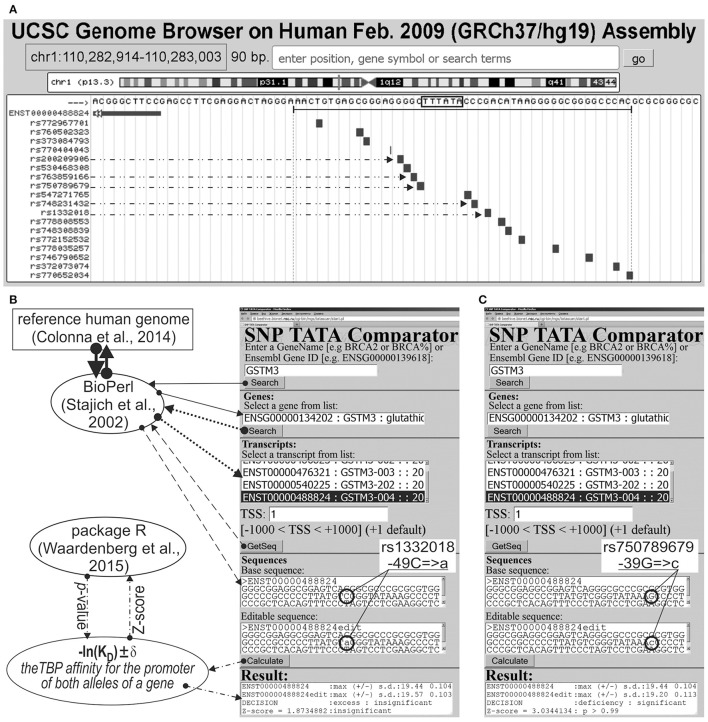
Known and candidate SNP markers of Alzheimer's disease (AD) near TBP-binding sites of the human GSTM3 gene promoter. **(A)** Unannotated SNPs (analyzed in this study) in the region [−70; −20] (where all proven TBP-binding sites (boxed) are located; double-headed arrow, ↔) of the human GSTM3 gene promoter taken from dbSNP (Sherry et al., 2001) using the UCSC Genome Browser (Haeussler et al., 2015). Dash-and-double-dot arrows: known and candidate SNP markers of sporadic AD are predicted by a significant change in the affinity of TBP for the human GSTM3 gene promoter. **(B,C)** The results from our Web service (Ponomarenko et al., 2015) for the two SNP markers of sporadic AD: known rs1332018 (Hong et al., 2009; Tan et al., 2013) and candidate marker rs750789679 near the known TBP-binding site (boxed) of the human GSTM3 gene promoter. Solid, dotted, and dashed arrows indicate queries for the gene list, list of transcripts of a certain gene, and DNA sequence of the promoter corresponding to the specified transcript by means of the BioPerl library (Stajich et al., 2002) of the reference human genome (Colonna et al., 2014), respectively. Dash-and-dot arrows: estimates of significance of the alteration of gene product abundance in patients with the minor allele (mut) relative to the norm (ancestral allele, wt) expressed as a Z-score using package R (Waardenberg et al., 2015). Circles indicate the ancestral (wt) and minor (mut) alleles of the SNP marker labeled by its dbSNP ID (Sherry et al., 2001).

We analyzed 626 SNPs retrieved from the dbSNP database, v.147 (Sherry et al., 2001), within [−70; −20] promoter regions of either 34 human genes containing SNP markers of hereditary diseases whose effects on TBP's binding to these promoters are clinically identified as described in our review (Ponomarenko et al., 2015), or five human genes—*MAPT, APP, PSEN1, PSEN2*, and *APOE*—associated with familial AD, which were taken from another article (Iwata et al., 2014). Figure 1A illustrates how we selected SNPs using the public Web service “UCSC Genome Browser” (Haeussler et al., 2015).

### Synthetic double-helical deoxyoligonucleotides (ODNs)

The ODNs identical to ancestral and minor alleles of the selected SNPs—rs563763767, rs33980857, rs34598529, rs33931746, rs33981098, rs35518301, rs1143627, rs72661131, rs7277748, and rs1800202—were synthesized and purified (BIOSYN, Novosibirsk, Russia).

### Preparation and purification of recombinant full-length human TBP

Recombinant full-length human TBP (native amino acid sequence) was expressed in *Escherichia coli* BL21 (DE3) cells transformed with the pAR3038-TBP plasmid (a generous gift from Prof. B. Pugh, Pennsylvania State University) as described by Pugh (1995) with two modifications: the IPTG concentration was 1.0 instead of 0.1 mM, and the induction time was 3 instead of 1.5 h (for more details, see Savinkova et al., 2013).

### Electrophoretic mobility shift assay (EMSA) under equilibrium conditions *in vitro*

The above ODNs were prepared by ^32^P labeling of both strands by means of T4 polynucleotide kinase (SibEnzyme, Novosibirsk) with subsequent annealing by heating to 95°C (at equimolar concentrations) and slow cooling (no less than 3 h) to room temperature. Equilibrium dissociation constants (K_D_) for the TBP–ODN complex were measured using a conventional protocol (Savinkova et al., 2013). It includes titration of a fixed amount of the above-mentioned TBP, 0.3 nM, with the increasing concentrations of the ODN to reach equilibrium, whose time was determined independently for each ODN in advance. The binding experiments were conducted at 25°C in a buffer consisting of 20 mM HEPES-KOH pH 7.6, 5 mM MgCl_2_, 70 mM KCl, 1 mM EDTA, 100 μg/ml BSA, 0.0.1% of NP-40, and 5% of glycerol. The TBP–ODN complexes were separated from the unbound ODN using an EMSA, and their concentrations were measured. These experimental data were input into the conventional software OriginPro 8, whose output was a K_D_ value expressed in nanomoles per liter, nM.

### Stopped-flow fluorescence measurements *in vitro* in real-time mode

The above ODNs identical to both ancestral 5′-cgcggcgctcTATATAAgtgggcagt-3′ and minor 5′-cgcggcgctcTATAgAAgtgggcagt-3′ alleles of the selected SNP rs1800202 were labeled at 5′ termini with fluorescent dyes TAMRA and FAM (BIOSYN, Novosibirsk, Russia). Combining a fixed concentration (0.1 μM) of ODNs with various concentrations (0.1, 0.2, 0.4, 0.6, 0.8, or 1.0 μM) of the above TBP, we analyzed six time-series of the fluorescence expressed in conventional units using high-resolution spectrometer SX.20 (Applied Photophysics, UK). These experimental data were input into the Dynafit software (Biokin, USA) whose output was the above K_D_ values (for more details, see Arkova et al., 2016).

### Cell culture, transfection, and reporter assays *ex vivo*

Cell line hTERT-BJ1 (human fibroblasts) was cultivated in a complete medium consisting of Dulbecco's modified Eagle's medium/Nutrient mixture F-12 Ham, supplemented with 10% (v/v) of fetal bovine serum (Sigma), penicillin (100 U/mL), and streptomycin (100 μg/mL; BioloT). The culture was maintained at 37°C in a humidified atmosphere containing 5% of CO_2_ until the desired level of confluence. The proximal core promoter 177 bp long containing either the ancestral allele or minor allele of the selected candidate SNP marker rs201381696 (5′-atcgggccgcTATAAGAggggcgggc-3′ or 5′-atcgggccgcTgTAAGAggggcgggc-3′, respectively) was cloned into the pGL 4.10 vector (Promega, USA) and cotransfected with pRL-TK using Screen Fect A (InCella) as described by Wolfe et al. (2002). Next, the cells were cultured in 6-well plates for 24 h. Luciferase activity was determined using the Dual-Luciferase Reporter Assay Kit (Promega). All the experiments were conducted in 11 independent replicates at 80–85% confluence.

### DNA sequence analysis

Figures 1B,C illustrates how we retrieved the promoter sequences containing these SNPs from the reference human genome (Colonna et al., 2014) using the BioPerl library (Stajich et al., 2002) via our publicly available Web service (Ponomarenko et al., 2015). Here, the “Base sequence” textbox contains the ancestral allele of a given promoter under study. Similarly, the “Editable sequence” textbox contains the minor allele of the analyzed SNP of this promoter handmade according to its description taken from the dbSNP database, v.147 (Sherry et al., 2001). These two DNA sequences constitute the input of the DNA sequence analysis algorithm whose detailed description and comprehensive formulae are in Supplementary File 1 “Supplementary Method.” Clicking on the “Calculate” button (Figures 1B,C) starts this algorithm's execution, whose output data will appear within the “Result” textbox.

This algorithm takes into account three steps of the TBP–promoter binding according to both prediction *in silico* (Ponomarenko et al., 2008) and detection *in vitro* (Delgadillo et al., 2009). These steps are (i) TBP slides along DNA (Coleman and Pugh, 1995; Karas et al., 1996; Ponomarenko et al., 1999) ↔ (ii) TBP stops at a TBP-binding site (Berg and von Hippel, 1987; Bucher, 1990) ↔ (iii) the TBP–promoter complex is fixed by DNA helix's bending to the 90° angle (Ponomarenko et al., 1997; Flatters and Lavery, 1998; Powell et al., 2002).

On this basis, we estimated statistically whether the alteration of the TBP binding affinity for the minor alleles of these promoters is insignificant (Figure 1B) or significant (Figure 1C) in comparison with the ancestral ones. To this end, we applied only one Fisher's Z-score (i.e., the ratio of the difference to the root of the sum of the squares of the standard deviations) using the standard statistical package R (Waardenberg et al., 2015). Our heuristic interpretation of our predictions of either significant over- or underexpression of the human genes is shown in italics in the second rightmost column of the tables in this paper with the word “*hypothetically”* in front of these interpretations.

### Keyword search in the PubMed database

After that, we discarded SNPs whose effects were insignificant; otherwise, we handmade a two-step keyword search in the PubMed database (NCBI Resource Coordinators, 2015). Figure S1 (hereinafter: see Supplementary File 2) depicts how we found sporadic AD as a comorbidity of the hereditary diseases associated clinically with the human genes containing the analyzed SNP near the TBP-binding sites of promoters of these genes.

In Figure S1, two boxes (dashed lines) depict the primary keyword search for these comorbidities whose known biochemical markers match the predicted significant alterations of the gene expression caused by the SNP under study. Additionally, we did a secondary manual keyword search for co-occurrence of sporadic AD and the hereditary disease clinically associated with the gene containing the SNP being considered (a box outlined with a dotted line). According to the positive or negative outcome of this additional keyword search, we either predicted the SNP being tested as a candidate SNP marker of sporadic AD as a comorbidity or discarded them. These are clinical data found during our manual keyword search, with the corresponding references in the rightmost column of the tables in this paper that are also shown in italics and marked with the phrase “(*this work*)” in front of these references found.

### Statistical comparison between the human genes associated with the familial AD and the entire human genome as a whole

Finally, we analyzed five human genes—*MAPT, APP, PSEN1, PSEN2*, and *APOE*—by the same method because these genes have been clinically most reliably implicated in familial AD (Iwata et al., 2014). Instead of the non-statistical secondary keyword search described above, we statistically compared these AD-associated genes and the entire human genome as a whole in terms of the ratio of SNP-caused over- or underexpression of the human genes in this comparison using the standard package Statistica (Statsoft™, Tulsa, USA). For this purpose, we used the conventional approach to estimation of natural selection pressure on these genes using analogies with the evolution speed rated by the ratio of frequencies of transitions and transversions (Kimura, 1980) and an adaptive evolution mode detected by the ratio of frequencies of synonymous and non-synonymous substitutions within the protein-coding DNA regions (Li et al., 1985).

## Results and discussion

### Candidate SNP markers of sporadic AD near TBP-binding sites of the human gene promoter associated with hereditary susceptibility to cancers

We employed our experimentally completely verified publicly available Web service (Ponomarenko et al., 2015) to analyze all SNPs in [−70; −20] proximal promoter regions of the human genes containing the known SNP markers (of hereditary diseases) that alter TBP's binding to promoters of these genes. Let us review in detail only one gene whose SNP marker is clinically associated with sporadic AD and, on this basis, we will do the same briefly for all the other uncovered genes.

**The human ***GSTM3*** gene** codes glutathione S-transferase μ3 and contains a clinically proven SNP marker (rs1332018) of sporadic AD (Hong et al., 2009) and renal cell carcinomas (Tan et al., 2013). According to the empirical data from a western blot (Wb) (Tan et al., 2013), this SNP reduces the *GSTM3* gene expression because it damages the binding site for an unknown tissue-specific transcription factor (rather than the binding site for the ubiquitous TBP) (Table 1). As one can see in Figure 1B, line “Decision” of the text box “Results” contains the label “insignificant,” which means that the prediction of our Web service (Ponomarenko et al., 2015) is consistent with the independent empirical data mentioned above (Tan et al., 2013). Near this known biomedical SNP marker, we found two unannotated SNPs (rs200209906 and rs750789679), which can also reduce the *GSTM3* gene expression by damaging the TBP-binding site (hereinafter: according to our predictions, see Figure 1 and Table 1). That is why, we propose them as two candidate SNP markers of sporadic AD (Hong et al., 2009) and renal cell carcinomas too (Table 1). In addition, we found two unannotated SNPs (rs748231432 and rs763859166), which, on the contrary, can increase the TBP–promoter affinity as two candidate SNP markers reducing the risk of sporadic AD (Table 1). Table S1 (hereinafter: see Supplementary File 3) shows the results of our non-statistical cross-validation of the candidate SNP markers of sporadic AD and renal cell carcinomas using the keyword search on the Web regarding co-occurrence of these diseases. Deficiency in Wilms' tumor suppressor is clinically associated with both (Lovell et al., 2003). In addition, we found a review on the state of US health between 1990 and 2010 (Murray et al., 2013), which lists both sporadic AD and renal cell carcinomas among the eight diseases whose age-standardized number of years of life lost to premature mortality increases year after year. These clinical cases and retrospective review show evidence supporting the known SNP markers of renal cell carcinomas as candidate SNP markers for sporadic AD (Table 1).

**Table 1:** Known and candidate SNP markers of sporadic AD near TBP-binding sites in the promoter of the human genes associated with susceptibility to cancers.

***Gene* (*OMIM ID*)**	**dbSNP rel. 147 or see (Ref)**	**5′ flank**	**wt** **mut**	**3′ flank**	**K**_**D**_ **, nM**					**Known diseases (known SNP markers) or *hypothetical disease* (*candidate SNP markers*)**	**(Ref) or (*this work*)**
					**wt** **mut**	**Δ**	**Z**	**α**	**ρ**		
*GSTM3 (138390)*	rs1332018	ccccttatgt	**c** *a*	gggtataaag	**4** 3	=	2		E	“**c**” associated with AD and renal cell carcinomas (Wb: TF-binding site damaged, not TBP-binding site)	Hong et al., 2009; Tan et al., 2013
	rs200209906	gtataaagcc	**c** t,a	ctcccgctca	**3.6** 4.3	↓	2		E	(*hypothetically*) *higher risks of AD and renal cell carcinoma*	(*this work*)
	rs750789679	cgggtataaa	**g** c	cccctcccgc	**3.6** 4.5	↓	3	10^−2^	C		
	rs748231432	cccttatgtc	**g** c,t	ggtataaagc	**3.6** 3.0	↑	3	0.05	D	(*hypothetically*) *lower risks of AD and renal cell carcinomas*	
	rs763859166	gggtataaag	**c** t	ccctcccgct	**3.6** 2.9	↑	3	10^−2^	C		
*IL1B* (*147720*)	rs1143627	ttttgaaagc	**c** *t*	ataaaaacag	**5** *2*	↑	15	10^−6^	A	Liver cancer; gastric cancer, gastric ulcer, and chronic gastritis in Hp-infection; non-small cell lung cancer, Graves' disease, recurrent major depression, greater body fat; (*hypothetically*) *greater Aβ-plaque clearance and blood-brain barrier damage in AD*	Ponomarenko et al., 2015, (*this work*) Rivera-Escalera et al., 2014; Wang et al., 2014
	rs549858786	tgaaagccat	**a** *t*	aaaacagcga	**5** 7	↓	8	10^−6^	A	(*hypothetically*) *lower Aβ-plaque clearance and smaller blood-brain barrier damage in AD*	
*DHFR* (*126060*)	rs10168	ctgcacaaat	**g** *a*	gggacgaggg	**15** *9*	↑	9	10^−6^	A	Resistance to methotrexate treatment of leukemia and, also, (*hypothetically*) *lower risk of AD*	Al-Shakfa et al., 2009
	rs750793297	tgcacaaatg	**g** *t*	ggacgagggg	**15** *13*	↑	3	10^−2^	C	(*hypothetically*) *lower risk of AD and resistance to methotrexate treatment of leukemi*	(*this work*) Banka et al., 2011; Turnaev et al., 2016
	rs766799008	ctgcacaaat	**a** *g*	tggggacgag	**15** *19*	↓	3	10^−3^	B	(*hypothetically*) *higher risk of AD and greater effectiveness of methotrexate-based therapy for leukemia in children*	
	rs764508464	ctgcacaaat	**a** –	tggggacgag	**15** *37*	↓	17	10^−6^	A		
	rs754122321	ctcgcctgca	**c** *g*	aaatggggac	**15** *25*	↓	9	10^−3^	B		

*Hereinafter, ancestral (wt) and minor (mut) alleles; K_D_, dissociation constant of TBP–DNA (Savinkova et al., 2013); Δ, a change: excess (↑), deficit (↓), norm (=); α = 1–p, significance {where p value is shown in Figure 1}; ρ, heuristic ranks of candidate SNP markers varying in alphabetical order from the “best” (A) to the “worst” (E); 18 bp, the 18-bp deletion g_−72_ggcggacatacatatac_−54_ (the human CETP gene); 25 bp, the 25-bp deletion g_−63_cggcccgtttctcgggcttcgggc_−39_ (the human PSEN1 gene); Aβ, β-amyloid; AD, Alzheimer's disease; ALS, amyotrophic lateral sclerosis; AS, atherosclerosis; DM, diabetes mellitus; EMSA, electrophoretic mobility shift assay; Hb, hemoglobins; Hp, Helicobacter pilori; LUC, luciferase reporter assay; TF, transcription factor; T1D, type 1 diabetes; Wb: western blot*.

**The human ***IL1B*** gene** (interleukin 1β) promoter has an SNP marker (rs1143627) causing overexpression, which is clinically associated with virus-induced cancers in the liver and in the stomach as well as with non-small cell lung cancer, with Graves' disease, major recurrent depression, obesity, gastric ulcer, and chronic gastritis (for review, see Ponomarenko et al., 2015) (Table 1). According to our primary keyword search (hereinafter: Figure S1: Supplementary File 2), Rivera-Escalera et al. (2014) detected the interleukin-1β-mediated amyloid plaque clearance in both familial and sporadic AD, and Wang et al. (2014) observed interleukin-1β-induced blood-brain barrier disruption complicating both familial and sporadic AD (Table 1). As for the secondary keyword search results (hereinafter: Figure S1: Supplementary File 2), we found three works on negative correlations between sporadic AD and both non-small cell lung cancer (Grinberg-Rashi et al., 2009; Akushevich et al., 2013) and Graves' disease (Yoshimasu et al., 1991), whereas seven studies (Ades and Lejoyeux, 1994; Reynolds et al., 1995; Bopp-Kistler et al., 1999; Wang et al., 2003; Kountouras et al., 2007; Ge and Sun, 2011; Kim and Choi, 2015) are in favor of co-occurrence between this disease and the remaining pathologies associated with rs1143627 (Table S1). Thus, the known clinical SNP marker rs1143627 may also be a candidate SNP marker of both the interleukin-1β-mediated amyloid plaque clearance and induced blood-brain barrier damage in both familial and sporadic AD as a complication of major recurrent depression, greater body fat, gastritis, gastric ulcer, and gastric, and liver cancers rather than lung cancer or Graves' disease (Table S1). Finally, we found an annotated SNP (rs549858786) near a known clinical SNP marker, rs1143627, and predicted by means of our Web service (Ponomarenko et al., 2015) that this SNP can cause a deficiency of interleukin-1β (Table 1). For the reasons above, we propose rs549858786 as a candidate SNP marker of both low clearance of amyloid plaques and smaller blood-brain barrier damages in both familial and sporadic AD with respect to the norm (Table 1).

**The human ***DHFR*** gene** (dihydrofolate reductase) contains a known clinical SNP marker (rs10168; within a promoter), which can cause overexpression of this gene associated with resistance to methotrexate treatment in children with acute lymphoblastic leukemia (Al-Shakfa et al., 2009) as one can see in Table 1. Our primary keyword search produced a retrospective clinical review (Banka et al., 2011), which is associating both DHFR deficiency and poor cognitive function and dementia in the elderly (e.g., sporadic AD) with one another. In addition, phosphatidylinositol-binding clathrin assembly protein (PICALM) contributes to both leukemia and sporadic AD pathogenesis (Xiao et al., 2012), whereas methotrexate can cause long-term changes in astrocytes (Gregorios et al., 1989) and increase amyloid toxicity (Kruman et al., 2002) in sporadic AD according to our secondary keyword search results. Accordingly, we predicted that the known clinical SNP marker rs10168 can also be a candidate SNP marker of a lower risk of sporadic AD. Finally, we found four unannotated SNPs, rs750793297, rs766799008, and rs764508464, and rs754122321, which, on the contrary, can cause DHFR deficiency and, thus, may serve as candidate SNP markers of a high risk of sporadic AD (Banka et al., 2011) (Table 1).

Table 1 and Table S1 together indicate that the regulatory SNP markers of both Graves' disease and lung cancer may be candidate SNP markers of a lower risk of sporadic AD because the etiologies are equally unrelated to that of sporadic AD. In addition, the high risk is predictable by regulatory SNP markers of at least renal, blood, liver, and gastric cancers because of common players in their pathogenesis, such as: Wilms' tumor suppressor, phosphatidylinositol-binding clathrin assembly protein, and antibodies against proteins of *Helicobacter pylori* and hepatitis C virus, which are mimics β-amyloid peptides, respectively. Thus, if someone carrying a minor allele of SNPs listed in Tables 1 and S1 has a reduced risk of renal, blood, liver, and gastric cancers (rather than lung cancer) in his/her childhood, adolescence and adulthood, then he/she additionally is at a lower risk of sporadic AD in his/her old age.

### Candidate SNP markers of sporadic AD near TBP-binding sites of the human gene promoter associated with hereditary immune diseases

**The human ***CETP*** gene** codes for cholesterol ester transfer protein; in its promoter, there is a known SNP marker of a lower risk of atherosclerosis due to hyperalphalipoproteinemia following CETP deficiency caused by 18-bp deletion G_−72_GGCGGACATACATATAC_−54_ upstream of the transcription start site (Plengpanich et al., 2011). According to our keyword search results (Table 2), this gene contains another known SNP marker (rs1800775) of CETP deficiency increasing three-fold the risk of sporadic AD (Rodriguez et al., 2006). Within the same core-promoter, we found three unannotated SNPs (rs17231520, rs757176551, and rs569033466), which can, on the contrary, cause overexpression of *CETP* (Table 2) and thereby reduce the risk of sporadic AD. As for our secondary keyword search, it resulted in the article (Birkenhager and Staessen, 2004) showing that AS and sporadic AD coexist, correlate, enhance each other clinically and that both AS and sporadic AD can be caused by a fast response to acute infection as a replacement of deadly stress with long-term near-norm complications progressing slowly (Lathe et al., 2014), e.g., β-amyloid plaques (Table S1). Within the limitations of the pathogenesis of both AS and sporadic AD when they can be caused by acute infection as it was suggested by the article (Lathe et al., 2014), we predicted that all four above-mentioned SNPs of the human *CETP* gene core promoter are the candidate SNP markers of sporadic AD (Table 2).

**Table 2:** Known and candidate SNP markers of sporadic AD near TBP-binding sites of promoters of the human genes associated with the hereditary immune system-related diseases.

***Gene* (*OMIM ID*)**	**dbSNP] rel. 147 or see [Ref]**	**5′ flank**	**wt** **mut**	**3′ flank**	**K**_**D**_ **, nM**					**Known diseases (known SNP markers) or *hypothetical disease* (*candidate SNP markers*)**	**(Ref) or (*this work*)**
					**wt** **mut**	**Δ**	**Z**	**α**	**ρ**		
*CETP (118470)*	DEL-51(18 bp) Plengpanich et al., 2011	cgtgggggct	**18bp** −	gggctccagg	**4** *7*	↓	7	10^−6^	A	Hyperalphalipoproteinemia that prevents atherosclerosis and (*hypothetically*) *higher risk of AD* (*rs1800775 of CETP [down] increases risk of AD*)	Plengpanich et al., 2011 (*this work*), Rodriguez et al., 2006
	rs17231520	ggggctgggc	**g** *a*	gacatacata	**4** *2*	↑	10	10^−6^	A	(*hypothetically*) *lower risk of AD*	
	rs569033466	atacatatac	**g** *a*	ggctccaggc	**4** *3*	↑	4	10^−3^	B		
	rs757176551	catatacggg	**c** *g*	tccaggctga	**4** *2*	↑	*10*	10^−6^	A		
*MMP12* (*601046*)	rs2276109	gatatcaact	**a** *g*	tgagtcactc	**11** *14*	↓	3	10^−2^	C	Lower risk of psoriasis, systemic sclerosis, and asthma	Ponomarenko et al., 2015, (*this work*), Walker et al., 2006; Ito et al., 2007
	rs572527200	gatgatatca	**a** *g*	ctatgagtca	**11** *14*	↓	3	10^−2^	C	(*hypothetically*), *lower risk of AD (MMP12 excess provokes inflammatory response whose result is neuronal cell loss)*	
*MBL2* (*154545*)	rs72661131	tctatttcta	**t** *c*	atagcctgca	**2** *4*	↓	12	10^−6^	A	Variable immunodeficiency, preeclampsia, stroke	Ponomarenko et al., 2015, (*this work*), Sjolander et al., 2013
	rs562962093	atctatttct	**a** *g*	tatagcctgc	**2** *5*	↓	15	10^−6^	A	(*hypothetically*) *higher risk of AD*	
	rs567653539	tttctatata	**g** *a*	cctgcaccca	**2** *1*	↑	12	10^−6^	A	(*hypothetically*) *lower risk of AD*	
*SOD1* (*147450*)	rs7277748	ggtctggcct	**a** *g*	taaagtagtc	**2** ***7***	↓	17	10^−6^	A	Amyotrophic lateral sclerosis, (*hypothetically*), *higher risks of Aβ oligomerization and memory loss in AD*	Niemann et al., 2007, (*this work*), Murakami et al., 2011

**The human ***MMP12*** gene** codes for matrix metallopeptidase 12 and carries a known SNP marker (rs2276109) that reduces this gene's expression and, thus, lowers the risks of psoriasis, systemic sclerosis, and chronic asthma in children and in smokers (for review, see Ponomarenko et al., 2015) as one can see in Table 2. In this table, we show an unannotated SNP (rs572527200) located near this known biomedical SNP marker, which can also reduce this gene's expression and, hence, be a candidate SNP marker for the same diseases. Our primary keyword search yielded empirical data (Walker et al., 2006; Ito et al., 2007) showing that in contrast, the MMP12 excess provokes an inflammatory response whose consequence can be neuronal cell loss in both familial and sporadic AD. In addition (Table S1), our secondary keyword search showed several lines of evidence cross-validating that psoriasis, systemic sclerosis, and asthma coexist with sporadic AD in terms of the common drugs against them (e.g., Hori et al., 2015) as well as their common biomarkers (e.g., Wei et al., 2010; Yokoyama et al., 2016). Within limitations of the assumption that the effectiveness of common drugs and biomarkers in different diseases may be evidence of some relation between them, we propose rs2276109 and rs572527200 as candidate SNP markers of a reduced risk of sporadic AD (Table 2).

**The human ***MBL2*** gene** (soluble mannose-binding lectin 2) contains a known SNP marker (rs72661131) of variable immunodeficiency, preeclampsia, and stroke as one can see in our review (Ponomarenko et al., 2015). This SNP reduces expression of this gene according to our Web service's prediction (Ponomarenko et al., 2015). Our primary keyword search pointed to the clinical data (Sjolander et al., 2013) on the MBL2 deficiency as a significant biochemical marker of sporadic AD. Near this SNP rs72661131, we found two unannotated SNPs (rs562962093 and rs567653539) that can respectively reduce and elevate the *MBL2* expression as well as increase and decrease risks of this disease (Table 2). Our secondary keyword search revealed that there are drugs against both variable immunodeficiency and sporadic AD (Puli et al., 2012); the search also showed that human genome region 10q22 is associated with both preeclampsia and sporadic AD (van Dijk et al., 2010), whereas a natural marine product diet reduces the risks of both stroke and sporadic AD (Choi and Choi, 2015), as one can see in Table S1. This supporting information allows us to suggest three candidate SNP markers (rs72661131, rs562962093, and rs567653539) of sporadic AD (Table 2).

**The human ***SOD1*** gene** (soluble superoxide dismutase 1): its promoter contains a known SNP marker (rs7277748) of familial amyotrophic lateral sclerosis (ALS) because this SNP causes *SOD1* excess (Niemann et al., 2007). Using a primary keyword search, we learned that this *SOD1* excess is a biochemical marker of both β-amyloid oligomerization and memory loss in sporadic AD (Murakami et al., 2011) (Table 2). As for the co-occurrence of sporadic AD and ALS, our secondary keyword search identified two works (Hamilton and Bowser, 2004; Rusina et al., 2007) and, additionally, one more article (Di Matteo and Esposito, 2003) showing that a diet enriched in antioxidants reduces the risks of both diseases (Table S1). For all these reasons, the known SNP marker (rs7277748) of ALS can additionally be a candidate SNP marker of sporadic AD (Table 2).

Bringing the above findings together, we can suppose that some SNP markers of the immune-system-related diseases in adulthood can additionally be candidate SNP markers of sporadic AD in the elderly (Table 2 and Table S1). Consequently, when someone carrying minor alleles of SNPs shown in Table 2 and Table S1 is at a lower risk of the corresponding autoimmune diseases in his/her childhood, adolescence and adulthood, then he/she furthermore is at a lower risk of sporadic AD in his/her old age.

### Candidate SNP markers of sporadic AD near TBP-binding sites of the human gene promoter associated with hereditary blood diseases

**The human ***HBB*** and ***HBD*** genes** (β- and δ-hemoglobin, respectively) are the most thoroughly characterized in terms of clinical regulatory SNP markers, seven of which (Table 3: rs34500389, rs33981098, rs33980857, rs34598529, rs33931746, rs397509430, and rs35518301) reduce the affinity of the TBP for promoters of these genes and cause both thalassemia and malaria resistance (Martiney et al., 1996). Near these SNPs, we found three unannotated SNPs (rs63750953, rs281864525, and rs34166473) that can also cause hemoglobin deficiency with the same manifestation (Table 3). Using a primary keyword search, we found that both the homozygote-related deficiency and excess of hemoglobin are associated with sporadic AD and cognitive decline (Ferrer et al., 2011; Shah et al., 2011) because hypoxia is a biomarker of these diseases (Shang et al., 2015) and hemoglobin and β-amyloid peptides aggregate with one another in plaques (Chuang et al., 2012). In contrast, a retrospective review (Fallahzadeh et al., 2009) indicates that the corresponding heterozygotes protect humans against sporadic AD. In addition, our secondary keyword search showed (Table S1) that there are both common diets (Aruoma et al., 2010) and drugs (Dwyer et al., 2009) against both thalassemia and sporadic AD and that manzamine-type alkaloids are drugs against both malaria and sporadic AD (Rao et al., 2006). Finally, the race-biased susceptibility to sporadic AD is associated with the *APOE* gene (apolipoprotein E) whose SNPs can prevent malaria (Fujioka et al., 2013). Accordingly, we predict homozygotes of the above-mentioned SNPs (which can cause hemoglobin deficiency) to be candidate SNP markers of a higher risk of sporadic AD, whereas the corresponding heterozygotes are candidate SNP markers of the lower risk of this disease.

**Table 3:** Known and candidate SNP markers of sporadic AD near TBP-binding sites in the promoter of the human genes associated with hereditary blood diseases.

***Gene (OMIM ID)***	**dbSNP rel. 147 or see (Ref)**	**5′ flank**	**wt** **mut**	**3′ flank**	**K**_**D**_ **, nM**					**Known diseases (known SNP markers) or *hypothetical disease (candidate SNP markers)***	**(Ref) or *(this work)***
					**wt** **mut**	**Δ**	**Z**	**α**	**ρ**		
*HBB (141900)*	rs397509430	gggctgggca	**t** −	atacaacagt	**5** *29*	↓	34	10^−6^	A	Malaria resistance, Cooley's anemia (thalassemia), and *(hypothetically) in homozygotes, high risk of AD (both under- and over-expression of either HBB or HBD genes are associated with an increased risk of AD and more rapid cognitive decline (i.e., abnormal Hb abundance correlates with both AD and cognitive decline), whereas Aβ and Hb aggregate with each other near traumatic vascular injury in the brain); heterozygote protects from AD*	Martiney et al., 1996, (*this work*), Fallahzadeh et al., 2009; Ferrer et al., 2011; Shah et al., 2011; Chuang et al., 2012; Shang et al., 2015
	rs33980857	gggctgggca	**t** *a,g,c*	atacaacagt	**5** *21*	↓	27	10^−6^	A		
	rs34598529	ggctgggcat	**a** *g*	aaagtcaggg	**5** *18*	↓	24	10^−6^	A		
	rs33931746	gctgggcata	**a** *g,c*	aagtcagggc	**5** *11*	↓	14	10^−6^	A		
	rs33981098	agggctgggc	**a** *g,c*	taaaagtcag	**5** *9*	↓	10	10^−6^	A		
	rs34500389	cagggctggg	**c** *a,t,g*	ataaaagtca	**5** *6*	↓	3	10^−2^	C		
*HBD (142000)*	rs35518301	caggaccagc	**a** *g*	taaaaggcag	**4** *8*	↓	11	10^−6^	A		
*HBB (141900)*	*rs63750953*	ctgggcataa	**aa** −	gtcagggcag	**5** *8*	↓	*9*	*10^−6^*	A		
	*rs281864525*	tgggcataaa	**a** c	gtcagggcag	**5** *7*	↓	*7*	*10^−6^*	A		
*HBD (142000)*	rs34166473	aggaccagca	**t** *c*	aaaaggcagg	**4** *8*	↓	*18*	*10^−6^*	A		
*TPI1 (190450)*	rs1800202	gcgctctata	**t** g	aagtgggcag	**1** 4	↓	17	10^−6^	A	Hemolytic anemia, neuromuscular disease	Ponomarenko V. et al., 2016 (*this work*), Tajes et al., 2014
	rs781835924	cgcggcgctc	**t** c	atataagtgg	**1** 2	↓	10	10^−6^	A	*(hypothtically) high risk of AD (TPI1 deficiency correlates with AD); lower neuron viability and greater learning and memory deterioration in AD*
*APOA1 (107680)*	−35a→c Matsunaga et al., 1999	tgcagacata	**a** *c*	ataggccctg	**3** ***4***	↓	5	10^−6^	A	Fatty liver; *(hypothetically) lower risk of AD (another SNP rs670 increasing APOA1 expression elevates risks of AD and cognitive decline) except in the case of fatty liver*	Matsunaga et al., 1999, *(this work)*, Vollbach et al., 2005

**The human ***TPI1*** gene** encodes triosephosphate isomerase 1 and carries a known SNP marker (rs1800202) of hemolytic anemia and neuromuscular diseases manifesting themselves as a deficiency of this enzyme (for review, see Ponomarenko et al., 2015) (Table 3). Near this known biomedical SNP marker (rs1800202), we found an unannotated SNP (rs781835924), which can also reduce this gene's expression and thus manifest itself in the same manner. According to our primary keyword search results (Table 3), lower neuron viability and more severe learning and memory deterioration are the other manifestations of the TPI1 deficiency, which elevates the risk of sporadic AD (Tajes et al., 2014). In addition (Table S1), our secondary keyword search produced a retrospective review of 120 clinical cases suggesting that hemolytic anemia elevates the risk of death in both familial and sporadic AD, which cause not only intellectual impairment but also deteriorated physical conditions, motor dysfunction, and malnutrition (Ueki et al., 1995). We also found the article (Calne and Eisen, 1990) on the common etiological mechanism underlying both sporadic AD and neuromuscular diseases. Therefore, we predict that two candidate SNP markers (rs1800202 and rs781835924) increase the risk of sporadic AD in the elderly.

**The human ***APOA1*** gene** (apolipoprotein A-I) contains the −35A→C substitution reducing the expression of this gene, which is a known SNP marker of fatty liver in childhood (Matsunaga et al., 1999). Our primary keyword search revealed another known SNP marker (rs670) increasing the *APOA1* expression that elevates the risk of sporadic AD and cognitive decline (Vollbach et al., 2005). Next, our cross-validating secondary keyword search yielded an article (Tong et al., 2009) on nitrosamine as a common biochemical marker of both non-alcoholic fatty liver and sporadic AD, whereas the human *APOA1* gene is a homolog of the human *APOE* gene (Calandra and Tarugi, 1989), which is a key player in sporadic AD pathogenesis. Hence, we predicted this known SNP marker of fatty liver as a candidate SNP marker of the lower risk of sporadic AD except in the case of fatty liver (Table 3).

Summarizing all the above, we can conclude that many of known and candidate SNP markers of hereditary blood diseases may also be candidate SNP markers of sporadic AD in the elderly (Table 3 and Table S1). Therefore, someone who has a lower risk of hereditary blood diseases in his/her childhood, adolescence and adulthood, is also at a lower risk of sporadic AD in his/her old age.

### Candidate SNP markers of sporadic AD near TBP-binding sites of the human gene promoter associated with hereditary cardiovascular diseases

**The human ***THBD*** gene** (thrombomodulin) carries a known SNP marker (rs13306848) of thrombosis (Le Flem et al., 1999). This SNP damages a tissue-specific transcription factor (TF)-binding site near the known ubiquitous TBP-binding site (rather than this TBP-binding site itself) according to the results of a luciferase (LUC) reporter assay (Le Flem et al., 1999) (Table 4). Nevertheless, we found an unannotated SNP (rs568801899) able to cause thrombomodulin deficiency and, on the contrary, a review (Borroni et al., 2002) stating that the TBHD excess is a biochemical marker of sporadic AD (Table 4). In addition, the secondary search revealed (Table S1) that fibrinogen excess (Cortes-Canteli et al., 2010) contributes to both thrombosis and sporadic AD, whereas RU-505 is a drug effective against both (Ahn et al., 2014). This information allows us to assume that both rs13306848 and rs568801899 can be candidate SNP markers of a low risk of sporadic AD (Table 4).

**Table 4:** Known and candidate SNP markers of sporadic AD near TBP-binding sites of the promoter of human genes associated with hereditary cardiovascular diseases.

***Gene (OMIM ID)***	**dbSNP rel. 147 or see (Ref)**	**5′ flank**	**wt** **mut**	**3′ flank**	**K**_**D**_ **, nM**					**Known diseases (known SNP markers) or *hypothetical disease (candidate SNP markers)***	**(Ref) or (*this work*)**
					**wt** **mut**	**Δ**	**Z**	**α**	**ρ**		
*THBD (188040)*	rs13306848	agggagggcc	**g** *a*	ggcacttata	**2** 2	=	1		E	Thrombosis (LUC: TF-binding site damaged, not TBP-binding site)	Le Flem et al., 1999, *(this work)*, Borroni et al., 2002
	rs568801899	caatccgagt	**g** *a*	tgcggcatca	**45** 70	↓	6	10^−6^	A	*(hypothetically) low risk of AD whose biochemical marker is thrombomodulin excess*	
*F3 (134390)*	rs563763767	ccctttatag	**c** *t*	gcgcggggca	**3** *2*	↑	6	10^−6^	A	Myocardial infarction; thrombosis; *(hypothetically) high risk of AD (F3 is in Aβ-plaques)*	Arnaud et al., 2000, *(this work)*, McComb et al., 1991
*F7 (613878)*	−33a→c Kavlie et al., 2003	ccttggaggc	**a** *c*	gagaactttg	**53** *62*	↓	3	10^−2^	C	Moderate bleeding	Kavlie et al., 2003, *(this work)*,
	rs749691733	agaactttgc	**c** *t*	cgtcagtccc	**53** *66*	↓	4	10^−3^	B	*Higher risk of AD (another SNP rs6046 reduces F7 activity that was associated with AD)*	Barber et al., 2015
	*rs367732974*	aactttgccc	**g** *a*	tcagtcccat	**53** *47*	↑	2	0.05	D	*lower risk of AD (F7 excess is negatively associated with AD)*	
	*rs549591993*	gcccgtcagt	**c** *a*	ccatggggaa	**53** *25*	↑	13	10^−6^	A		
	rs777947114	agagaacttt	**g** *a*	cccgtcagtc	**53** *19*	↑	*19*	10^−6^	A		
	rs770113559	gtcacccttg	**g** *a*	aggcagagaa	**53** *41*	↑	*5*	10^−6^	A		
	rs754814507	cctcccccat	**c** *t*	cctctgtcac	**53** *45*	↑	*3*	10^−3^	B		
*GJA5 (121013)*	rs10465885	caactaagat	**g** a	tattaaacac	**3** 3	=	1		E	Arrhythmia, cardiovascular events (LUC: TF-binding site damaged, not TBP-binding site; mut 200% of wt),	Wirka et al., 2011, *(this work)*, Mo et al., 2014
										*(hypothetically) lower risk of AD development after stress*	
	rs35594137	gaggagggaa	**g** a	gcgacagata	**6** 6	=	0		E	Arrhythmia, cardiovascular events (LUC: TF-binding site damaged, not TBP-binding site; mut 50% of wt)	Firouzi et al., 2004, *(this work)*, Mo et al., 2014
	rs587745372	ggcgacagat	**a** t	cgattaaaaa	**6** 7	↓	*3*	10^−3^	B	*(hypothetically) GJA5 deficiency increases risk of AD development after stress*	(*this work*), Mo et al., 2014

**The human *F3* gene** encodes thromboplastin (synonyms: tissue factor, coagulation factor III) and contains a known SNP marker (rs563763767) of myocardial infarction and thromboembolism caused by F3 excess (Arnaud et al., 2000). Our primary keyword search revealed (Table 4) that thromboplastin is present within β-amyloid plaques in both familial and sporadic AD (McComb et al., 1991). In addition to the above associations between this disease and thrombosis shown in Table S1, our secondary keyword search revealed donepezil to be a drug effective against both myocardial infarction and sporadic AD (Arikawa et al., 2011) as well as common pathways of their pathogenesis (Licastro et al., 2011). With this in mind, we propose SNP marker rs563763767 of blood diseases as a candidate SNP marker of sporadic AD.

**The human ***F7*** gene** encodes proconvertin (synonym: coagulation factor VII), where an undocumented SNP (the a→c substitution at position -35 upstream of the transcription start site of this gene) has been clinically implicated in moderate bleeding caused by proconvertin deficiency (Kavlie et al., 2003). As one can see in Table 4, the unannotated SNP rs749691733 can also cause an F7 deficiency. Five other unannotated SNPs (rs367732974, rs549591993, rs777947114, rs770113559, and rs754814507) can elevate this gene's expression. Using our primary keyword search, we learned that another SNP (rs6046) is associated with both F7 deficiency and sporadic AD, whereas the F7 excess negatively correlates with this disease (Barber et al., 2015). Finally, our secondary keyword search produced the article (Nagasawa et al., 2014) on co-occurrence of sporadic AD and microbleed, as well as a retrospective review (Cordonnier and van der Flier, 2011) suggesting that brain microbleeds are intimately involved in the pathogenesis of both familial and sporadic AD (Table S1). Thus, we predicted two candidate SNP markers (–35a→c and rs749691733) in favor and five candidate SNP markers (rs367732974, rs549591993, rs777947114, rs770113559, and rs754814507) against sporadic AD (Table 4).

**The human ***GJA5*** gene** (connexin 40; synonym: gap junction protein α5) contains two biomedical SNP markers (rs10465885 and rs35594137) of arrhythmia and cardiovascular events (Firouzi et al., 2004; Wirka et al., 2011), which reduce this gene's expression via disruption of the binding site for an unknown transcription factor rather than the TBP-binding site according to a luciferase reporter assay, LUC (Table 4). Near these known SNP markers, we found an unannotated SNP (rs587745372), which can also reduce this gene's expression and, thus, points to the same diseases (Table 4). Using a primary keyword search, we found an original work on the connexin 40 deficiency as a biochemical marker of sporadic AD development after stress (Mo et al., 2014). In addition, our secondary keyword search yielded the article (Zulli et al., 2008) on co-occurrence of arrhythmia and sporadic AD, as well as retrospective reviews on the very close relation between sporadic AD, arrhythmia, and cardiovascular events in terms of their common medications (Winslow et al., 2011) and risk factors (Stone, 2008) in the elderly. For these reasons, we predict that three candidate SNP markers (rs10465885, rs35594137, and rs587745372) are associated with a higher risk of sporadic AD.

Overall, considering all the above results, we propose that several SNP markers of hereditary cardiovascular diseases may also be candidate SNP markers of sporadic AD in the elderly (Table 4 and Table S1). Thus, the reduced risks of cardiovascular disease in childhood, adolescence, and adulthood can reduce the risk of sporadic AD in the elderly.

### Candidate SNP markers of sporadic AD near TBP-binding sites of the human gene promoter associated with hereditary hormone-related diseases

**The human ***GH1*** gene** for somatotropin (synonym: growth hormone 1) contains a biomedical SNP marker (rs11568827) of short stature (Horan et al., 2003) caused by underexpression of this gene because this SNP damages the binding site for an unknown transcription factor rather than the TBP-binding site according to results of an electrophoretic mobility shift assay (EMSA) stature (Horan et al., 2003) (Table 5). Our primary keyword search produced the article (Malek et al., 2009) on the somatotropin-based treatment of some complications of sporadic AD (Turnaev et al., 2016). In addition, our secondary keyword search yielded a lot of articles on the negative correlation between this disease and the human stature, the most interesting of which (in our opinion) states that stature maximization in both childhood and adolescence as the integral indicator of health can minimize (or even delay) dementia in the elderly (1892 cases) (Beeri et al., 2005) (Table S1). Therefore, we predict that this known SNP marker (rs11568827) of short stature can also be a candidate SNP marker of the higher risk of sporadic AD.

**Table 5:** Known and candidate SNP markers of sporadic AD near TBP-binding sites of the promoter of the human genes associated with the hereditary hormone-related diseases.

***Gene***	**dbSNP rel. 147 or see (Ref)**	**5′ flank**	**wt** **mut**	**3′ flank**	**K**_**D**_ **, nM**					**Known diseases (known SNP markers) or *hypothetical disease (candidate SNP markers)***	**(Ref) or [*this work*]**
					**wt** **mut**	**Δ**	**Z**	**α**	**ρ**		
*GH1 (139250)*	rs11568827	aggggccagg	**g** −	tataaaaagg	1.5 *1.4*	=	1		E	Short stature (EMSA: unknown TF-binding site lost, not TBP-binding site)	Horan et al., 2003, (*this work*), Malek et al., 2009; Turnaev et al., 2016
	rs796237787	gaaggggcca	**g** −	ggtataaaaa							
	rs768454929	agggtataaa	**a** *c*	agggcccaca	1.5 *2.6*	↓	7	10^−6^	A	*(hypothetically) higher risks of AD and apparent effectiveness of somatotropin as a drug for AD but weaker spatial cognition in the elderly with AD*	
	rs761695685	gccagggtat	**a** *g*	aaaagggccc	1.5 *5.8*	↓	19	10^−6^	A		
	rs774326004	ccagggtata	**a** *t*	aaagggccca	1.5 *0.9*	↑	7	10^−6^	A	*(hypothetically) lower risk of AD and apparent effectiveness of somatotropin as a drug for AD but stronger spatial cognition in the elderly with AD*	
	rs777003420	aaggggccag	**g** *t*	gtataaaaag	1.5 *1.3*	↑	3	0.05	D		
*INS (176730)*	rs5505	agatcactgt	**c** *t*	cttctgccat	**53** *44*	↑	4	10^−3^	B	Type 1 diabetes (T1D) after neonatal diabetes mellitus (DM)	Landrum et al., 2014, *(this work)*, Picone et al., 2015
	rs563207167	tcagccctgc	**c** *t*	tgtctcccag	**53** *44*	↑	4	10^−3^	B	*(hypothetically) low risk of AD (insulin excess reduces both Aβ abundance and cell death)*	
	rs11557611	gatcactgtc	**c** *t*	ttctgccatg	**53** *60*	↓	2	0.05	D	*(hypothetically) high risk of AD*	
*GCG (138030)*	rs183433761	gctggagagt	**a** *g*	tataaaagca	0.9 *1.6*	↓	17	10^−6^	A	*(hypothetically) both glucagon deficiency and hyperleptinemia in urban children can elevate risk of AD in the elderly*	Chadaeva et al., 2016, *(this work)*, Tezapsidis et al., 2009; Calderon-Garciduenas et al., 2015
	rs757035851	tatataaaag	**cag** −	tgcgccttgg	0.9 *1.1*	↓	3	10^−3^	B		
*LEP (164160)*	rs200487063	tgatcgggcc	**g** *a*	ctataagagg	**4** *2*	↑	6	10^−6^	A		
	rs34104384	ccgctataag	**a** *t*	ggggcgggca	**4** *3*	↑	4	10^−2^	C		
	rs201381696	tcgggccgct	**a** *g*	taagaggggc	**4** *12*	↓	17	10^−6^	A	*(hypothetically) higher risk of AD where Aβ aggregates can cause hypothalamic leptin signaling dysfunction leading to early body weight deficits; AD treatment involves nutritional assessments and dietary measures; there is leptin replacement therapy for AD in case of leptin deficiency and weight loss*	

Two base pairs away from this known SNP marker (rs11568827), we found an unannotated SNP (rs796237787), which also represents a deletion of G with the same effect on the same gene expression (Table 5). That is why, we also propose rs796237787 as a candidate SNP marker of the higher risk of sporadic AD. In addition, we found two unannotated SNPs (rs768454929 and rs761695685) reducing the *GH1* gene expression as is the case for the known SNP marker rs11568827 described above. Thus, we nominate them as candidate SNP markers of the higher risk of sporadic AD too. Finally, near the known SNP marker rs11568827, we detected the other two unannotated SNPs (rs777003420 and rs774326004), which can cause the somatotropin excess and hence serve as candidate SNP markers of the lower risk of sporadic AD (Table S1).

**The human ***INS*** gene** encodes insulin, and its promoter contains a known SNP marker (rs5505) of type 1 diabetes (T1D) after neonatal diabetes mellitus (Landrum et al., 2014) caused by this gene overexpression (Table 5). Our primary keyword search revealed that an insulin excess reduces the β-amyloid abundance (Picone et al., 2015), which can reduce the risk of sporadic AD (Table 5). Near this known SNP marker, we found unannotated rs563207167 and rs11557611, which can cause hyper- and hypoinsulinemia, respectively, as well as a lower and higher risk of sporadic AD, respectively (Table 5). In addition, our secondary keyword search produced the article (Barrou et al., 2008) on the co-occurrence of diabetes and sporadic AD (Table S1), as well as two more interesting works (in our opinion), which are classifying both familial and sporadic AD as either type 3 diabetes or brain diabetes (Narasimhan et al., 2014; Kandimalla et al., 2016). That is why we propose these three candidate SNP markers of sporadic AD (Table 5).

**The human genes ***GCG*** and ***LEP***** encode glucagon and leptin (hunger hormone and obesity hormone, respectively), in whose promoters none of biomedical SNP markers have been found yet. In our previous work (Chadaeva et al., 2016), we made a computer-based prediction: we proposed three obesity-related candidate SNP markers (rs183433761, rs757035851, and rs201381696) of the significant deficiency of these hormones as well as the other two candidate SNP markers (rs200487063 and rs34104384) of significant overexpression of the *LEP* gene. In addition, we selectively verified this prediction in our experiments *in vitro* using EMSA (Chadaeva et al., 2016) and, independently, in cultures of the human cells transfected with the pGL 4.10 vector (Promega, USA) containing the reporter gene *LUC* (luciferase) (Chadaeva et al., 2016). In this work, our primary keyword search revealed that both glucagon deficiency and hyperleptinemia in urban children elevate the risk of sporadic AD in the elderly (Calderon-Garciduenas et al., 2015) and there is a leptin-based treatment of this disease (Tezapsidis et al., 2009) (Table 5). Finally, our cross-validating secondary keyword search showed a significant positive correlation between obesity in childhood, adolescence, or adulthood on the one hand and sporadic AD on the other hand (Renvall et al., 1993), whereas both body weight and fat loss regardless of diets and lifestyles are biomarkers of progression of this disease (Pedditizi et al., 2016) (Table S1). Thus, we predict five additional candidate SNP markers (rs183433761, rs757035851, rs200487063, rs34104384, and rs201381696) of sporadic AD, as shown in Table 5.

Looking through Table 5 and Table S1, we can expect that one can find candidate SNP markers of AD in the elderly among SNP markers of the hormone-related diseases of childhood, adolescence, and adulthood. Generally, one can see in Tables 1–5 and Table S1 taken all together that sporadic AD in the elderly seems to be a comorbidity of many hereditary blood, immune, hormone, and cardiovascular diseases. Hence, a healthy lifestyle, with a reduced risk of such diseases in childhood, adolescence, and adulthood can reduce the risk of sporadic AD in old age.

### Candidate SNP markers near TBP-binding sites within promoters of the human genes associated with the familial AD

**The human ***MAPT*** gene** (microtubule-associated protein tau; synonyms: neurofibrillary tangle protein, protein phosphatase 1) has no known biomedical SNP markers within its promoters, but we first found two candidate SNP markers (rs553179073 and rs11872014) of deficient expression of this gene and another marker (rs374878846) of its overexpression (Table 6). Using a primary keyword search, we learned that a MAPT excess may shift cellular gene networks toward AD-related cytopathogenesis (Saman et al., 2014) and enhance neuroinflammation (Wes et al., 2014) and neuronal loss (Wirths and Bayer, 2010). In contrast, a MAPT deficiency can increase survival in both familial and sporadic AD (Ittner et al., 2010), prevent memory deficits, and have a neuroprotective effect (Maphis et al., 2015) although it is also a biochemical marker of long-term depression (Kimura et al., 2013) and frontotemporal degeneration (Papegaey et al., 2016). Within these limits, we can predict three candidate SNP markers (rs553179073, rs11872014, and rs374878846) of familial AD.

**Table 6:** Candidate SNP markers near TBP-binding sites within promoters of the human genes associated with familial AD.

***Gene (OMIM ID)***	**dbSNP rel. 147 or see (Ref)**	**5′ flank**	**wt** **mut**	**3′ flank**	**K**_**D**_ **, nM**					***Hypothetical disease, which could be associated with a candidate SNP marker (Figure S1: Supplementary File 2)***	**References**
					**wt** **mut**	**Δ**	**Z**	**α**	**ρ**		
*MAPT (157140)*	rs374878846	accttctgcc	**g** a	ccgccaccac	**61** 55	↑	2	0.05	D	*(hypothetically) higher risks of AD, neuroinflammation in AD; neuronal loss*	*(this work)*, Wirths and Bayer, 2010; Saman et al., 2014; Wes et al., 2014
	rs553179073	ccgctgccac	**c** t	gcccaccttc	**61** 68	↓	2	0.05	D	*(hypothetically) higher survival in AD, prevention of memory deficits and neuroprotective effects but can be involved in long-term depression and frontotemporal degeneration*	*(this work)*, Ittner et al., 2010; Maphis et al., 2015; Papegaey et al., 2016
	rs11872014	acactcctca	**g** a	aacttatcct	**10** 13	↓	2	0.05	D		
*APP (104760)*	rs200621906	ggggtgggcc	**g** a	gatcagctga	**91** 56	↑	10	10^−6^	A	*(hypothetically) higher risk of AD (seven-fold APP-overexpressing mice of the APP23 strain are one of the widely accepted murine models of AD)*	*(this work)*, Sturchler-Pierrat et al., 1997
	rs536423638	cgggctccgt	**c** t	agtttcctcg	**68** 24	↑	19	10^−6^	A		
	rs558863815	gactcgcctg	**g** a	ctctgagccc	**109** 91	↑	4	10^−3^	B		
	rs759517529	actggctgaa	**g** a	aaagtgacaa	**36** 30	↑	4	10^−3^	B		
	rs756747509	ccctgcctca	**a** g	gtaacaattg	**10** 12	↓	3	10^−3^	B	*(hypothetically) higher risk of cognitive deficits without AD-like anatomical changes (APP^−/−^ mice)*	*(this work)*, Zhang et al., 2016
	rs561135968	aagaaaatcc	**t** a	acaaaaggaa	**6** 10	↓	7	10^−6^	A		
*APOE (107741)*	rs762555354	cccacctcg	**g** t	actgggggct	**58** 21	↑	15	10^−6^	A	*(hypothetically) lower risk of AD via reduced growth of Aβ plaques*	*(this work), Bien-Ly et al., 2012*
	rs758661090	gcgagactgg	**g** c	actgagatgg	**54** 48	↑	2	0.05	D		
	rs769448	gagatggaac	**g** c	ggcggtgggg	**54** 46	↑	3	10^−2^	C		
	rs758379972	ggggagccct	**a** g	taattggaca	**5** 16	↓	16	10^−6^	A	*(hypothetically) higher risk of AD via Aβ accumulation*	*(this work)*, Ohman et al., 1996
*PSEN1 (104311)*	rs201362083	ctcccctcct	**c** t	cgtgggccgg	**107** 58	↑	13	10^−6^	A	*(hypothetically) higher risk of AD via death of postmitotic neurons after injury, but lower risk of AD due to an anti-apoptotic protective effect in neurons after apoptosis induction*	*(this work)*, Vezina et al., 1999; Prat et al., 2002
	rs202209472	ggccgccaac	**g** a	acgccagagc	**107** 55	↑	13	10^−6^	A		
	rs1800839	ccgccaacga	**c** t	gccagagccg	**107** 72	↑	8	10^−6^	A		
	rs199959804	ggtggagaga	**g** a	attccgggga	**51** 35	↑	7	10^−6^	A		
	rs563558461	ggccccgccc	**c** t	cttcctcctg	**96** 78	↑	4	10^−3^	B		
	rs752158054	accaggaggg	25 bp −	gcggccgggt	**51** 83	↓	9	10^−6^	A	*(hypothetically) higher risk of AD with age-dependent onset (double knockout PSEN1^−/−^PSEN2^−/−^ murine model), but lower risk of AD due to PSEN1-deficiency inhibits Aβ-plaque formation and corrects hippocampal long-term potentiation*	*(this work)*, Dewachter et al., 2002; Aoki et al., 2009
	rs530970418	gccccgcccc	**c** g	ttcctcctgg	**96** 78	↓	4	10^−3^	B		
	rs772984560	aaacagtatt	**t** c	ctatacagtt	**3** 6	↓	7	10^−6^	A		
	rs796710298	gtatttctat	**t** c	cagttgctcc	**3** 6	↓	10	10^−6^	A		
*PSEN2 (600759)*	rs761796296	tgtttcattt	**c** t	gtgtgtgttg	**11** 9	↑	6	10^−6^	A	*(hypothetically) higher risk of AD*	(*this work*), Gamliel et al., 2002
	rs556146702	cgtggcctgg	**g** t	cgggcgtggg	**352** 220	↑	9	10^−6^	A		
	rs544497401	cccagtggac	**g** a	agggaacgcg	**81** 41	↑	11	10^−6^	A		
	rs758016212	ggggccccag	**t** c	ggacgaggga	**81** 117	↓	6	10^−6^	A	*(hypothetically) higher risk of AD with age-dependent emergence (double PSEN1^−/−^PSEN2^−/−^mice); AD-like neurodegeneration and lung tumor development (PSEN2^−/−^mice); age-dependent hemorrhage and pulmonary fibrosis (PSEN1^+/−^PSEN2^−/−^mice)*	*(this work)*, Herreman et al., 1999; Chen et al., 2008; Aoki et al., 2009; Yun et al., 2014
	rs564994558	gggccccagt	- g	ggacgaggga	**81** 102	↓	4	10^−3^	B		
	rs201944966	agagccggtt	**t** c	ctgttagcag	**25** 32	↓	4	10^−3^	B		

**The human ***APP*** gene** encodes β-amyloid precursor protein. Promoters of this gene contain no known biomedical SNP markers yet, but we for the first time identified four candidate SNP markers (rs200621906, rs536423638, rs558863815, and rs759517529) of the significant overexpression of this gene and two candidate SNP markers (rs756747509 and rs561135968) of the significant deficiency of β-amyloid peptides (Table 6). Our primary keyword search showed (Table 6) that both over- (Sturchler-Pierrat et al., 1997) and underexpression (Zhang et al., 2016) of the *APP* gene increase the familial AD risk because of β-amyloid aggregation (Sturchler-Pierrat et al., 1997) and cognitive deficits without AD-like anatomical changes in the brain (Zhang et al., 2016), respectively. Accordingly, we propose six candidate SNP markers (rs200621906, rs536423638, rs558863815, rs759517529, rs756747509, and rs561135968) of the higher risk of familial AD.

**The human ***APOE*** gene** (apolipoprotein E) contains no known biomedical SNP markers supported by clinical data within its promoters. Nevertheless, for the first time, we found three candidate SNP markers (rs762555354, rs758661090, and rs769448) of the significant *APOE* overexpression and another one (rs758379972) of significant *APOE* underexpression (Table 6). According to our primary keyword search results, the APOE excess reduces the risk of this disease (Ohman et al., 1996) and *vice versa* (Bien-Ly et al., 2012) (Table 6). Accordingly, we suggest four candidate SNP markers (rs762555354, rs758661090, rs769448, and rs758379972) of familial AD, its complications, and comorbidities (Table 6).

**Human genes ***PSEN1*** and ***PSEN2***** encode presenilins 1 and 2, respectively, in whose promoters there are no biomedical SNP markers, but we for the first time uncovered eight candidate SNP markers (rs201362083, rs202209472, rs1800839, rs199959804, rs563558461, rs761796296, rs556146702, and rs544497401) of a significant excess of presenilins and seven candidate markers (rs752158054, rs530970418, rs772984560, rs796710298, rs758016212, rs564994558, and rs201944966) of a significant presenilin deficiency (Table 6). Our primary keyword search revealed (Table 6) that both a presenilin excess (Vezina et al., 1999; Gamliel et al., 2002; Prat et al., 2002) and deficiency (Herreman et al., 1999; Dewachter et al., 2002; Chen et al., 2008; Aoki et al., 2009; Yun et al., 2014) elevate the risk of familial AD, whereas *PSEN1* underexpression can also reduce the risk of some complications of this disease. On the basis of these data, we propose 15 aforementioned SNPs as candidate markers of familial AD (Table 6).

In a final cross-validation test, we unexpectedly noticed that only a minority (12 of 28) of candidate SNP markers predicted here decrease expression of five genes associated with familial AD (Table 6) whereas the majority (35 of 56) of the candidate SNP markers can downregulate the human genes associated with other hereditary diseases (Tables 1–5 and Table S1). This difference is statistically significant (α < 0.05 according to binomial distribution). Moreover, we compared this minority of candidate SNP markers of underexpression of the human genes (associated with familial AD) with the commonly accepted whole-genome ratio 2:1 of the regulatory SNPs reducing vs. SNPs increasing affinity of the transcription factors for the human gene promoters; this ratio was identified by two independent groups of investigators (Kasowski et al., 2010; 1000 Genomes Project Consortium et al., 2012). This difference is statistically significant too (α < 0.01). This phenomenon may reflect the pressure of natural selection against the deficient expression of five genes *MAPT, APP, PSEN1, PSEN2*, and *APOE* associated with familial AD relative to both the whole genome and the genes associated with other hereditary diseases in humans. This finding indicates the higher robustness of these five genes on a genomewide scale.

This extraordinary robustness of genes *MAPT, APP, PSEN1, PSEN2*, and *APOE* is well-consistent with the manifestation of damage to these genes as familial AD only at the age of over 65. This is when the humans accumulate a number of pathologies that may not be associated with AD and whose interactions with these damaged genes allow these pathologies to manifest themselves as AD. Moreover, due to this robustness of these genes in childhood, adolescence, and adulthood, the human body can respond to deadly stressors (e.g., microbleeds, brain injury, or acute infection) by borderline slowly progressing pathologies (e.g., β-amyloid plagues) that develop only in the elderly as sporadic AD or other diseases of aging as noted previously (Lathe et al., 2014). In both above-mentioned cases, lowering the risk of the accumulated pathologies in childhood, adolescence, and adulthood corresponds to a lower risk of AD in old age. These matches between our predictions and the current knowledge about familial and sporadic AD support the candidate SNP markers predicted here.

### Verification procedures for the selected candidate SNP markers predicted here

**Figure 2:**
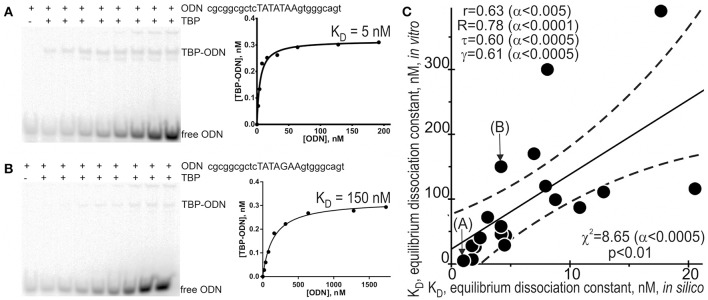
Experimental verification of the selected candidate SNP markers by an electrophoretic mobility shift assay (EMSA) *in vitro*. An example of electropherograms in the case of ancestral (**A**: norm, wild-type, wt) and minor (**B**: minor) alleles of the candidate SNP marker rs1800202 within the human *TPI1* gene promoter, which are accompanied by diagrams of experimentally measured values. **(C)** The significant correlations between the *in silico* predicted (X-axis) and *in vitro* measured (Y-axis) K_D_ values of the equilibrium dissociation constant of the [TBP–ODN] complex. Solid and dashed lines or curves denote the linear regression and boundaries of its 95% confidence interval, calculated using software STATISTICA (Statsoft™ USA). Circles denote the ancestral and minor alleles of the candidate SNP markers rs563763767, rs33980857, rs34598529, rs33931746, rs33981098, rs35518301, rs1143627, rs72661131, rs7277748, and rs1800202 being verified; r, R, τ, γ, χ^2^, and α are linear correlation, Spearman's rank correlation, Kendall's rank correlation, Goodman–Kruskal generalized correlation, χ^2^ test, and their significance, respectively; p is Fisher's exact test two marks (A) and (B) indicate by arrows two experimental magnitudes whose measurement procedures are shown in panels **A** and **B**, respectively.

Because different computational methods have their own advantages and disadvantages in predicting functional SNPs, to gain good-quality knowledge, a comparison between computer-based predictions and experimental data as an independent commonly accepted uniform platform is still needed in familial and sporadic ADs. That is why we selected some of the 89 candidate SNP markers predicted here—rs563763767, rs33980857, rs34598529, rs33931746, rs33981098, rs35518301, rs1143627, rs72661131, rs7277748, and rs1800202—and measured equilibrium dissociation constant K_D_ for the binding of human TBP to each of them using the conventional protocol of the EMSA *in vitro* (see section “Materials and Methods”). Figure 2 shows the results, namely: Figures 2A,B exemplify electropherograms and their graphical representations in the case of ancestral and minor alleles, respectively, of the candidate SNP marker rs1800202 within the human *TPI1* gene promoter. As one can see in Figure 2C, the predicted (axis X) and measured (axis Y) K_D_ values significantly correlate according to a number of statistical tests such as linear correlation (r), Spearman's rank correlation (R), Kendall's rank correlation (τ), Goodman–Kruskal generalized correlation (γ), and χ^2^ and Fisher's exact (p) tests. These robust correlations between our predictions and experimental data also support the candidate SNP markers (of familial and sporadic AD) predicted here.

**Figure 3:**
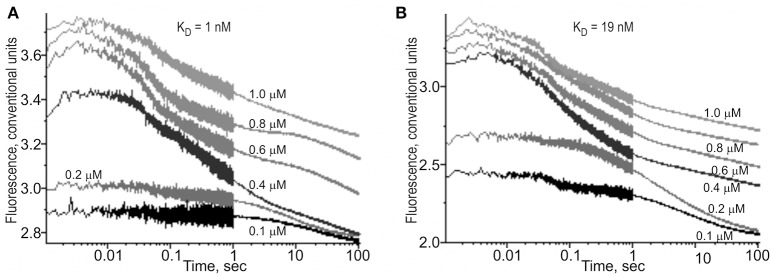
The kinetics of binding to and bending of the ODN identical to the selected SNP marker rs1800202. **(A)** The ancestral allele, ODN 5′-cgcggcgctcTATATAAgtgggcagt-3′. **(B)** The minor allele, ODN 5′-cgcggcgctcTATAgAAgtgggcagt-3′. ODN concentration was 0.1 μM. TBP concentration was varied between 0.1 and 1.0 μM as indicated near the corresponding curve of the time series. K_D_ values, **(A)** 1 nM and **(B)** 19 nM, were obtained as the output of the Dynafit software (Biokin, USA) after the corresponding time-series data were inputted into this software.

**Figure 4:**
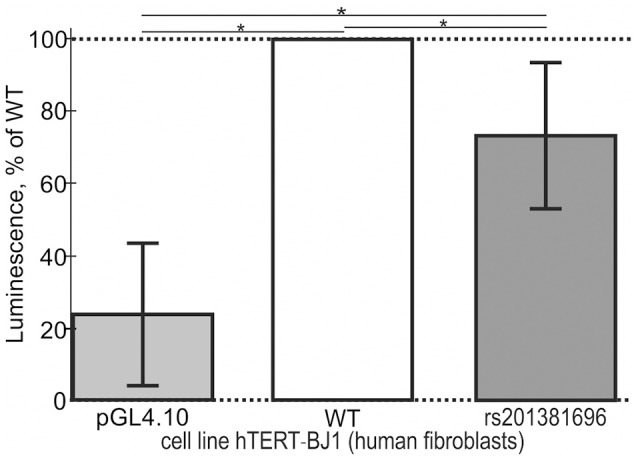
Cell culture verification of the selected candidate SNP marker rs201381696 in cell line hTERT-BJ1 (human fibroblasts) transfected with the pGL 4.10 vector carrying a reporter *LUC* gene. Experimental data: open bars, ancestral allele (wild type, WT); two gray bars shown to the right and left of the open bar, namely, minor allele (rs201381696) and the original vector pGL 4.10 (Promega, USA) without any insertions, respectively, served as an independent control and minor allele, respectively. The height of the bars and their error bars correspond to the mean estimates and boundaries of their 95% confidence intervals calculated from 11 independent replicates of measurements. Asterisks indicate a statistically significant difference at the confidence level of α < 0.05.

In addition to the above-mentioned widely used conventional EMSA, we applied two modern high-performance methods. Figure 3 shows the results of the stopped-flow fluorescence assay *in vitro* in real time on a high-resolution spectrometer SX.20 (Applied Photophysics, UK) in the case of the selected candidate SNP-marker rs1800202. As one can see, our prediction in silico that this SNP is manifested as reduced affinity of TBP binding to the human *TPI1* gene promoter (Table 3) is consistent with the presented experimental data. Finally, in the case of the selected candidate SNP-marker rs201381696 predicted in this work, Figure 4 shows the results obtained by *ex vivo* verification using the human cell line hTERT-BJ1 (human fibroblasts) cultured and transfected with the pGL 4.10 vector carrying a reporter LUC gene (Table 5). As the table shows, the reduced affinity of TBP binding to the minor allele of this SNP (relative to the ancestral allele), which was predicted by us (Table 5), fits the *ex vivo* manifestation of this SNP within the pGL 4.10 vector carrying a reporter LUC gene. These two experiments once again lend support to the candidate SNP markers (of familial and sporadic AD) predicted here.

This comparison between the computer-based predictions of the candidate biomedical SNP markers and proper experimental measurements as an independent commonly accepted uniform platform are the novelty of this work relative to other computer-based methods and tools in this active field of research.

### How to use candidate SNP markers (of familial and sporadic AD) predicted here

Currently, the OMIM database (Amberger et al., 2015) describes more than a hundred well-known SNP markers associated with either familial or sporadic AD, the majority of which are located within the protein-coding regions of the genes and, thus, fatally disrupt either structures or functions of the proteins encoded by these genes. That is why medications, diets, or lifestyles cannot correct the resulting molecular aberrations because these interventions can relieve only the symptoms of AD. In addition to these valuable biomedical genome-wide data, we predicted 89 candidate regulatory SNP markers of familial (Table 6) and sporadic (Tables 1–5) AD, whose pathogenic effects are limited only to under- and overexpression of the corresponding genes without changes in protein structure or function (Tables 1–6 and Table S1). Because the structures and functions of the affected proteins remain native, and only amounts of the proteins are altered, this aberration of protein concentrations may be correctable by medications, diets, and lifestyle changes that reduce the risk of both familial and sporadic AD, their complications, and comorbidities.

Because the TBP-binding site is the best-studied regulatory sequence within the human genome (Amberger et al., 2015), we focused our research attention on SNPs altering TBP's affinity for human gene promoters. Using our Web service (Ponomarenko et al., 2015), we analyzed 629 SNPs located within [−70; −20] proximal promoter regions of 39 human genes and found 89 candidate SNP markers of familial and sporadic AD (Tables 1–6). This finding does not mean that the remaining 533 SNPs cannot be candidate SNP markers of the same pathology because these SNPs may alter transcription factor-binding sites (e.g., rs1332018, rs13306848, rs10465885, rs35594137, rs11568827, and rs796237787). To analyze any of them, there are a number of publicly available Web services (for review, see Deplancke et al., 2016) whose research capabilities can be enhanced when they are used together with others, including our Web service (Ponomarenko et al., 2015).

The gist of this paper is that 25 candidate SNP markers located within promoters of the human genes associated with familial AD (Table 6) may cause this disease. By contrast, 56 candidate SNP markers of other hereditary diseases (Tables 1–5 and Table S1) can only be genomewide informative landmarks that can facilitate the choice of physicians regarding appropriate treatment (based on the genomic data from their patients). These 56 candidate biomarkers may also help a random person to choose his/her lifestyle in childhood, adolescence, or adulthood to minimize the risk of sporadic AD later in life. For example, here we predicted candidate SNP markers of a higher risk of sporadic AD in the elderly as a complication of stroke (rs72661131 and rs562962093), which can help someone with minor alleles of these SNPs to include some natural marine products into his/her diet minimizing the risks of both diseases (Choi and Choi, 2015). Similarly, due to the candidate SNP marker rs7277748 of sporadic AD as a comorbidity of amyotrophic lateral sclerosis (Table S1), someone with minor alleles of this SNP can reduce the risks of these diseases by switching to a diet enriched in antioxidants (Di Matteo and Esposito, 2003). (Of course, this is oversimplification: if amyotrophic lateral sclerosis could be prevented by increased consumption of antioxidants alone, the lives of numerous patients would be very easy.) In addition, using our predicted candidate SNP markers of sporadic AD as a comorbidity of short stature (rs11568827, rs796237787, rs768454929, and rs761695685), parents of children carrying minor alleles of these SNPs can choose a lifestyle maximizing their stature in childhood and adolescence as an integral indicator of their health. This approach may then minimize (or even eliminate) the risk of dementia at their old age (Beeri et al., 2005).

Finally, each candidate SNP marker of either familial or sporadic AD proposed here should be first verified using clinical protocols, including representative cohorts of the relevant groups of patients and healthy volunteers (as a control). After that, such SNP markers may be applicable to clinical practice. To help with this validation, we accompanied each predicted candidate SNP marker by our prediction of the equilibrium dissociation constant (K_D_) for the binding of human TBP to a 26-bp synthetic duplex DNA identical to the SNP in question; the constant is expressed in nanomoles per liter, nM (Tables 1–6).

Finally, according to a huge number of publications, it seems inevitable that many of these SNPs will not help to identify the candidate SNP marker worth pursuing for subsequent experimental research (e.g., verification using either clinical protocols or animal models of human diseases created using CRISPR/Cas9 protocols of animal genome editing *in vivo*, Holm et al., 2016). With this in mind, we set up heuristic prioritization of the candidate SNP markers predicted here using the statistical significance rates of Fisher's Z-tests in terms of heuristic rank ρ-values, which vary in alphabetical order from the “best” (A) to the “worst” (E) as shown in Tables 1–6. We hope that this prioritization will make it possible to more successfully select the most promising candidate SNP markers of AD for their verification using clinical protocols or CRISPR/Cas9 protocols of animal genome editing *in vivo*.

## Concluding remarks

As soon as the proposed 89 candidate SNP markers of familial and sporadic AD are validated by clinical protocols, these whole-genome landmarks may become interesting to the general population (may help to choose a lifestyle in childhood, adolescence, or adulthood reducing the risks of sporadic AD, its comorbidities, or complications in the elderly).

## Author contributions

LS, ID, ES, and DZ designed the study. IC compiled data. NK created and maintained the software operation. EK performed experiments. DR and PP analyzed data. MP wrote the manuscript.

### Conflict of interest statement

The authors declare that the research was conducted in the absence of any commercial or financial relationships that could be construed as a potential conflict of interest.

## Footnotes

^1^ http://beehive.bionet.nsc.ru/cgi-bin/mgs/tatascan/start.pl
