# Supplementary material for: Candidate SNP Markers of Familial and Sporadic Alzheimer's Diseases Are Predicted by a Significant Change in the Affinity of TATA-Binding Protein for Human Gene Promoters
Source: Front Aging Neurosci. 2017 Jul 20;9:231. doi: 10.3389/fnagi.2017.00231 (PMC5517495; doi:10.3389/fnagi.2017.00231)
Supplement: Supplementary file 1 [file Table1.PDF]

Supplementary Material

Candidate SNP markers of familial and sporadic Alzheimer's diseases are predicted by a significant change in the affinity of TATA-binding protein for human gene promoters

Petr Ponomarenko, Irina Chadaeva, Dmitry Rasskazov, Ekaterina Sharypova, Elena Kashina, Mikhail Ponomarenko*, Ludmila Savinkova, Nikolay Kolchanov

*** Correspondence:** Mikhail Ponomarenko (pon@bionet.nsc.ru)

Table S1: Co-occurrence of sporadic AD and hereditary diseases whose candidate SNP markers were predicted within this work.

Candidate SNP-marker	Hereditary diseases	Co-occurrence of sporadic AD and hereditary diseases whose SNP markers are being analyzed	(Reference)
rs1332018, rs200209906, rs750789679, rs748231432, rs763859166	renal cell carcinomas	sporadic AD and renal cell carcinomas are two among the only eight diseases whose age-standardized numbers of years of life lost to premature mortality increase year after year in the USA; deficiency of Wilms' tumor suppressor (*WT1* gene) is associated with both sporadic AD and renal cell carcinomas among elderly and pediatric patients;	(Murray et al., 2013; Lovell et al., 2003)
rs1143627, rs549858786	non–small cell lung cancer	significant negative correlation between sporadic AD and non–small cell lung cancer; sporadic AD and lung cancer is the rarest comorbidity pair in the elderly	(Grinberg-Rashi et al., 2009; Akushevich et al., 2013)
	Graves' disease	significant negative correlation between sporadic AD and Graves' disease	(Yoshimasu et al., 1991)
	recurrent major depression	the same antidepressant drugs of maintenance therapy are used during recurrent major depression and both familial and sporadic AD in the elderly;	(Reynolds et al., 1995)
	liver cancer	mitogen-activated protein kinase-related signaling pathways contribute to both sporadic AD and drug-resistant liver cancer; alcohol and drug abuse cause both liver cancer and sporadic AD in the elderly	(Kim, and Choi, 2015; Ades, and Lejoyeux, 1994)
	Hp-caused gastric cancer, gastric ulcer, chronic gastritis	sporadic AD significantly positively correlates with Hp infection and vitamin B12 deficiency caused by gastritis; Hp's amyloid-like Hpn protein links sporadic AD with gastritis, gastric ulcer, and gastric cancer; alcohol and drug abuse cause gastritis and sporadic AD in the elderly	(Ades, and Lejoyeux, 1994; Ge, and Sun, 2011; Kountouras et al., 2007; Bopp-Kistler et al., 1999)
rs183433761, rs757035851, rs200487063, rs34104384, rs201381696	obesity	significant positive correlation between sporadic AD and obesity, whereas both body weight and fat loss regardless of diets and lifestyle are biomarkers of progression of both familial and sporadic AD	(Renvall et al., 1993 Pedditizi et al., 2016)

Notes: Aβ, β-amyloid; AD, Alzheimer's disease; ALS, amyotrophic lateral sclerosis; AS, atherosclerosis; DM, diabetes mellitus; Hb, hemoglobins; Hp, *Helicobacter pylori;* T1D, type 1 diabetes.

Table S1: Continued

Candidate SNP-marker	Hereditary diseases	Co-occurrence of sporadic AD and hereditary diseases whose SNP markers are being analyzed	(Reference)
rs10168, rs750793297, rs766799008, rs764508464, rs754122321	leukemia	phosphatidylinositol-binding clathrin assembly protein (PICALM) affects both leukemia and AD pathogenesis	(Xiao et al., 2012)
	methotrexate treatment	methotrexate can cause long-term changes in astrocytes and increase Aβ-toxicity as complications in sporadic AD (murine model)	(Gregorios et al., 1989; Kruman et al., 2002)
CETP: DEL-51(18bp) (Plengpanich et al., 2011), rs17231520, rs569033466, rs757176551	athero-sclerosis (AS)	Co-occurrence of AS and sporadic AD, they correlate and enhance each other; both AS and sporadic AD can be caused by cholesterol 25-hydroxylase excess as a fast response to acute infection as a replacement of deadly stress with long-term borderline complications progressing slowly (e.g., *Aβ plaques*)	(Birkenhager, and Staessen, 2004; JA Lathe et al., 2014)
rs2276109, rs572527200	asthma	diets and drugs against both asthma and sporadic AD	(Hori et al., 2015)
	systemic sclerosis	ligands of peroxisome proliferator-activated receptor γ are drugs effective against both sporadic AD and systemic sclerosis	(Wei et al., 2010)
	psoriasis	there is a known SNP marker (rs2516049) of both sporadic AD and psoriasis	(Yokoyama et al., 2016)
rs72661131, rs562962093, rs567653539	variable immune-deficiency	human intravenous immunoglobulin is a treatment of variable immunodeficiency that may account for its beneficial effect in both familial and sporadic AD	(Puli et al., 2012)
	preeclam-psia	both preeclampsia and sporadic AD are mapped onto the human genome region 10q22 where the preeclampsia-associated *STOX1* gene (isoform A) promotes the growth of Aβ plaques	(van Dijk et al., 2010)
	stroke	natural marine diet prevents both stroke and sporadic AD	(Choi, and Choi, 2015)
rs397509430, rs33980857, rs34598529, rs33931746, rs33981098, rs34500389, rs35518301, rs63750953, rs281864525, rs34166473	Cooley's anemia (thalas-semia)	the functional food supplement fermented papaya preparation as a new promising treatment of both thalassemia and sporadic AD; drugs reducing Fe concentration can treat both thalassemia and sporadic AD	(Aruoma et al., 2010; Dwyer et al., 2009)
	malaria resistance	manzamine-type alkaloids as drugs against both malaria and sporadic AD; APOE (keynote gene of both familial and sporadic AG pathogenesis) prevents growth of malaria; race-biased susceptibility to sporadic AD	(Rao et al., 2006; Fujioka et al. 2013)
rs1800202, rs781835924	hemolytic anemia	hemolytic anemia elevates risk of death in both familial and sporadic AD, which cause not only intellectual impairment but also deterioration of physical condition, motor dysfunction, and malnutrition (120 cases)	(Ueki et al., 1995)
	neuro-muscular diseases	death of some neurons and deterioration of living conditions of their surviving neighbors are a common etiological mechanism of both sporadic AD and neuromuscular diseases	(Calne, and Eisen, 1990)

Table S1: Continued

Candidate SNP-marker	Hereditary diseases	Co-occurrence of sporadic AD and hereditary diseases whose SNP markers are being analyzed	(Reference)
rs7277748	amyotro-phic lateral sclerosis (ALS)	Co-occurrence of ALS and sporadic AD; a diet enriched in antioxidants prevents both ALS and sporadic AD	(Hamilton, and Bowser, 2004; Rusina et al., 2007; Di Matteo, and Esposito, 2003)
APOA1: –35a→c (Matsunaga et al., 1999)	fatty liver	nitrosamine is a common biochemical marker of both nonalcoholic fatty liver and sporadic AD; *APOA1* as a homolog of *APOE* (a key player in both familial and sporadic AD pathogenesis)	(Tong et al., 2009; Calandra, and Tarugi, 1989)
rs13306848, rs568801899	thrombosis	fibrinogen and Aβ bind to each other as a risk factor for both thrombosis and both familial and sporadic AD; drug RU-505 significantly alleviates thrombosis and cognitive deficits	(Cortes-Canteli et al., 2010; Ahn et al., 2014)
rs563763767	myocardial infarction	Donepezil is a drug effective against both sporadic AD and myocardial infarction; pathogeneses of sporadic AD and myocardial infarction share similarities	(Arikawa et al., 2011; Licastro et al., 2011)
F7: -33a→c (Kavlie et al., 2003), rs749691733, rs367732974, rs549591993, rs777947114, rs770113559, rs754814507	bleeding	Co-occurrence of sporadic AD and microbleeds; brain microbleeds are intimately involved in both familial and sporadic AD pathogenesis	(Nagasawa et al., 2014; Cordonnier, and van der Flier, 2011)
rs10465885, rs35594137, rs587745372	arrhythmia	Co-occurrence of arrhythmia and sporadic AD; arrhythmia is the most common complication of drugs for both familial and sporadic AD	(Zulli et al., 2008;Winslow et al., 2011)
	cardio-vascular events	risk factors for both sporadic AD and cardiovascular events are significantly overlapping	(Stone, 2008)
rs11568827, rs796237787, rs768454929, rs761695685, rs774326004, rs777003420	short stature	maximized stature in childhood and adolescence minimizes (or delays) dementia in the elderly (the retrospective review based on 1892 cases)	(Beeri et al., 2005)
rs5505, rs563207167, rs11557611	type 1 diabetes	co-occurrence of sporadic AD and diabetes in the elderly; AD is either type 3 diabetes or diabetes of the brain	(Barrou et al., 2008; Narasimhan et al., 2014; Kandimalla et al. 2016)
