# Supplementary material for: Candidate SNP Markers of Familial and Sporadic Alzheimer's Diseases Are Predicted by a Significant Change in the Affinity of TATA-Binding Protein for Human Gene Promoters
Source: Front Aging Neurosci. 2017 Jul 20;9:231. doi: 10.3389/fnagi.2017.00231 (PMC5517495; doi:10.3389/fnagi.2017.00231)
Supplement: Supplementary file 2 [file Presentation1.PDF]

frontiers

Supplementary Material

Candidate SNP markers of familial and sporadic Alzheimer's diseases are predicted by a significant change in the affinity of TATA-binding protein for human gene promoters

Petr Ponomarenko, Irina Chadaeva, Dmitry Rasskazov, Ekaterina Sharypova, Elena Kashina, Mikhail Ponomarenko*, Ludmila Savinkova, Nikolay Kolchanov

*** Correspondence:** Mikhail Ponomarenko (pon@bionet.nsc.ru)

Figure S1: A flow chart of the keyword search for sporadic AD as a comorbidity of hereditary diseases whose known and candidate SNP markers can alter TBP-binding sites in the human gene promoters.

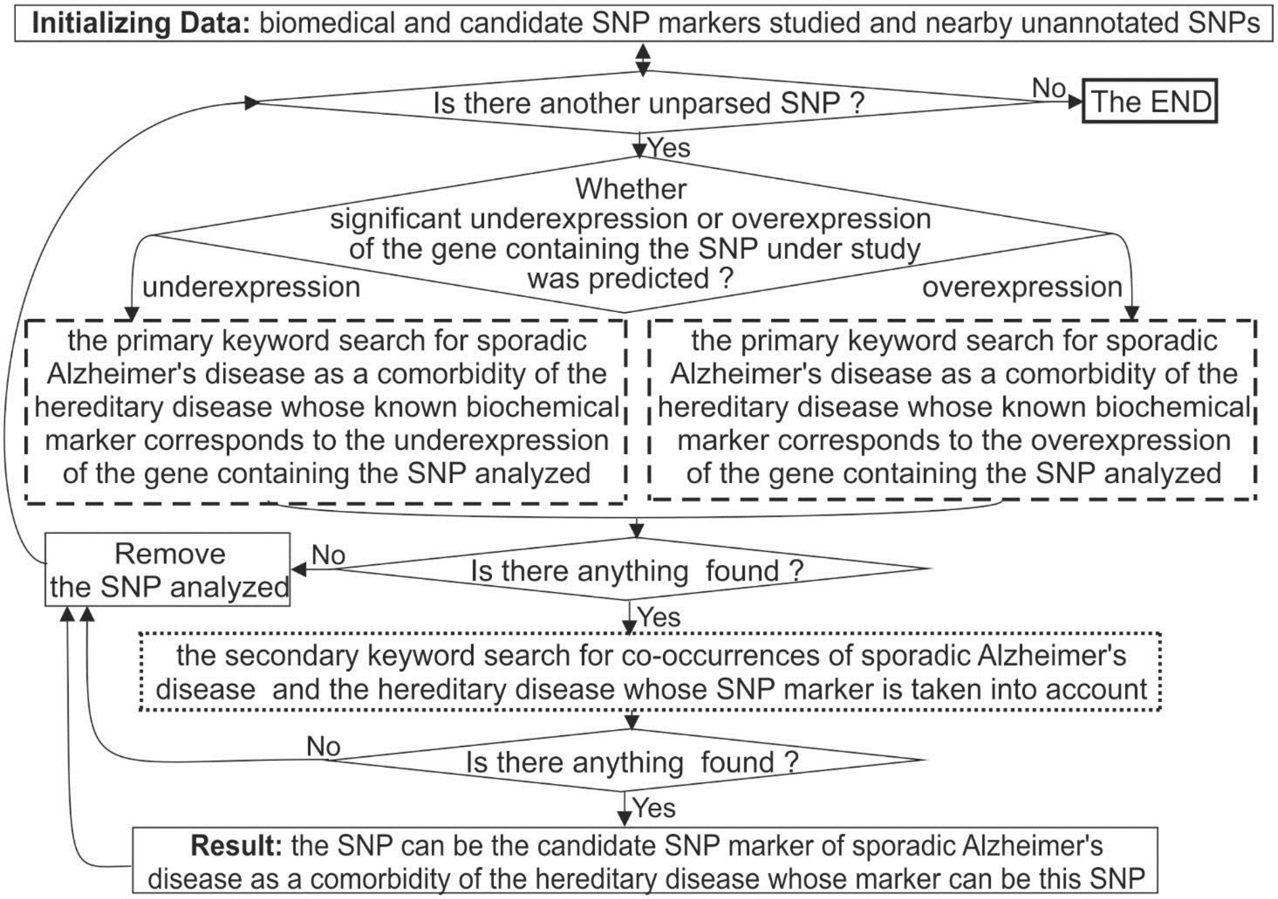

Legend. The boxes consisting of dashed lines depict the primary keyword search for sporadic AD as a comorbidity of the hereditary diseases being considered whose biochemical markers match the predicted significant alterations of the gene expression caused by the SNP under study. The dotted box depicts the secondary keyword search for co-occurrence of sporadic AD and the hereditary disease clinically associated with the SNP being.
