# Supplementary material for: Candidate SNP Markers of Familial and Sporadic Alzheimer's Diseases Are Predicted by a Significant Change in the Affinity of TATA-Binding Protein for Human Gene Promoters
Source: Front Aging Neurosci. 2017 Jul 20;9:231. doi: 10.3389/fnagi.2017.00231 (PMC5517495; doi:10.3389/fnagi.2017.00231)
Supplement: Supplementary file 3 [file Presentation2.PDF]

Supplementary Material

Candidate SNP markers of familial and sporadic Alzheimer's diseases are predicted by a significant change in the affinity of TATA-binding protein for human gene promoters

Petr Ponomarenko, Irina Chadaeva, Dmitry Rasskazov, Ekaterina Sharypova, Elena Kashina, Mikhail Ponomarenko*, Ludmila Savinkova, Nikolay Kolchanov

*** Correspondence:** Mikhail Ponomarenko (pon@bionet.nsc.ru)

1 A quantitative estimate of binding affinity of TATA-binding protein for a gene promoter within the 90-bp sequence immediately upstream of a given transcription start site

The input data are the DNA sequence $\{s_{-90}\ldots s_i\ldots s_{-1}\}$ of 90 bp in length, whose location is immediately upstream of the transcription start site (TSS, s_0) (where $s_i \in \{a, c, g, t\}$).

Within the framework of the linear approximation, we take into account the three-step molecular mechanism of the TATA-binding protein (TBP) binding to the [-70; -20] region of the eukaryotic gene promoters, which was first proposed as a hypothesis (Ponomarenko et al., 2008) and next observed empirically one year later (Delgadillo et al., 2009). We describe them (TBP slides along promoter DNA ↔ TBP stops at a potential TBP-binding site met ↔ the TBP-promoter complex isomerization due to DNA helix's bending to the 90° angle, which fixes this complex's conformation) as follows:

$$-\ln(K_D) = 10.9 - 0.2\ \{\ln(K_{SLIDE}) + \ln(K_{STOP}) + \ln(K_{BEND})\}, \qquad (1)$$

where 10.9 (ln units) is nonspecific TBP-DNA affinity (10^{-5} M), 0.2 is the stoichiometric coefficient, KSTOP is the equilibrium constant of the TBP stops at a TBP-binding site estimated heuristically using the maximal score value of Bucher's position-weight matrix, the commonly accepted criterion of the TATA-box as a canonical form of a TBP-binding site (Bucher, 1990);

$$\boldsymbol{ln(K_{STOP})} = \max_{(+),(-)\ \boldsymbol{DNA\ chains}} \left\{\sum\nolimits_{\boldsymbol{j=-1}}^{\boldsymbol{13}} \boldsymbol{w_{j;s_{i+j}}}\right\}; \qquad (2)$$

where w_{js} denotes an empirical estimate of the so-called "weight" of a given nucleotide $s \in \{a, c, g, t\}$ at j-th position of the TBP-binding site, whose values were published in the article (Bucher, 1990).

In Eq. (1), K_{SLIDE} is the equilibrium constant of the TBP sliding along DNA whose heuristic estimate can be described using the linear regression published in our article (Ponomarenko et al., 1999):

$$-\ln(K_{SLIDE}) = \mathrm{MEAN}_{15bp}\ \{0.8[TA] + 3.4MGW + 35.1\}, \qquad (3)$$

where [TA] is the abundance of dinucleotide TA; MGW is the minor groove width of the DNA helix (Karas et al., 1996); 0.8, 3.4, and 35.1 are linear regression coefficients taken from our original experimental data (Ponomarenko et al., 1999).

Supplementary Material

In Eq. (1), K_{BEND} is the equilibrium constant during the DNA helix bending; we estimated its value empirically as

$$-\ln(K_{BEND}) = MEAN_{TATA\text{-}box}\{0.9[TA, AA, TG, AG] + 2.5[TA, TC, TG] + 14.4\}, \quad (4)$$

where 0.9, 2.5, and 14.4 are linear regression coefficients calculated from our original experimental data (Ponomarenko et al., 1997); $MEAN_{TATA\text{-}box}$ is the means for both DNA strands of the TATA-box at the maximal score value of Eq. (2) (Bucher, 1990).

According to all the possible nucleotide substitutions, $s_j \rightarrow \xi$, at each position j within the 26-bp DNA window scanning the promoter DNA being analyzed (where $\xi \in \{a, c, g, t\}$), we estimated the standard deviation of the -ln[K_D] estimates (Eq. 1) as

$$\delta=[(\Sigma_{1\le i\le 26}\Sigma_{\xi\in \{a,c,g,t\}}[\ln(K_D(\{s_{i-13}\ldots\xi\ldots s_{i+12}\})/K_D(\{s_{i-13}\ldots{}_1 s_{i+j}\ldots s_{i+12}\})^2])/(3*26)]^{1/2} \quad (5)$$

Applying Eqs. (1–5) to the cases of two minor (mut) and ancestral (wt) alleles of a given gene promoter, we get $(-\ln(K_D^{(mut)}) \pm \delta_{(mut)})$ and $(-\ln(K_D^{(wt)}) \pm \delta_{(wt)})$, respectively, and next calculate Fisher's Z-score:

$$Z = abs[\ln(K_D^{(mut)}/K_D^{(wt)})]/[\delta^2_{(mut)}+\delta^2_{(wt)}]^{1/2}. \quad (6)$$

Using the statistical package R (Waardenberg et al., 2015)], we transform this Z-score value into the p-value of the probability rate of acceptance of the hypothesis "H_0: $K_D^{(mut)} \neq K_D^{(wt)}$" (where α = 1-p is the statistical significance level of this hypothesis' acceptance). At this statistically significant level $\alpha < 0.05$ (i.e., at $p > 0.95$), we made the final decision:

IF {*INEQUALITY* "$-\ln(K_D^{(mut)}) > -\ln(K_D^{(wt)})$" is statistically significant},

THEN {*DECISION* is "the minor allele of a given gene is overexpressed versus the ancestral one"};

ELSE [**IF** {*INEQUALITY* "$-\ln(K_D^{(mut)}) < -\ln(K_D^{(wt)})$" is statistically significant},

THEN {*DECISION* is "the minor allele of this gene is underexpressed versus the ancestral one"},]

OTHERWISE {*DECISION* is "alteration of the expression of this gene is insignificant"}.

This DECISION is shown in the text box "Result" of our publicly available Web service SNP_TATA_Comparator[1] (Ponomarenko et al., 2015), as illustrated in Figure 1(B),(C).

---

[1]http://beehive.bionet.nsc.ru/cgi-bin/mgs/tatascan/start.pl
